# Linking α-Fe_2_O_3_-modified zeolite, amendment structure, and PTE fractionation during municipal solid waste incineration bottom ash stabilization

**DOI:** 10.1039/d6ra06895b

**Published:** 2026-10-08

**Authors:** Thi Thu Thuy Nguyen, Truong Xuan Vuong, Thi Thu Ha Pham, Dinh Vinh Nguyen, Thi Viet Pham, Thi Giang Ngo, Anh Quoc Hoang, Minh Binh Tu, Thi Hue Nguyen, Xuan Hoa Vu, Tran Thu Trang, Xuan Thang Dam

**Affiliations:** a Faculty of Natural Sciences and Technology, TNU-University of Science Tan Thinh Ward Thai Nguyen City 24000 Vietnam xuanvt@tnus.edu.vn; b University of Science, Vietnam National University Hanoi, 19 Le Thanh Tong Hanoi 11000 Vietnam; c Institute of Science and Technology for Energy and Environment (ISTEE) A30 building, 18-Hoang Quoc Viet R., Nghia Do Hanoi Vietnam; d Institute of Science and Technology, TNU-University of Sciences Phan Dinh Phung Ward Thai Nguyen province 24000 Vietnam; e Faculty of Chemical Technology, Hanoi University of Industry (HaUI) No. 298 Cau Dien Street, Bac Tu Liem District Hanoi Vietnam

## Abstract

The environmental mobility of potentially toxic elements (PTEs) in municipal solid waste incineration (MSWI) bottom ash is strongly influenced by their partitioning among labile and less labile solid-phase fractions rather than by total concentrations alone. Although Fe-modified zeolites have been investigated for PTE stabilization, how Fe_2_O_3_ loading influences the evolution of amendment structure and PTE fractionation during curing remains insufficiently resolved. Here, α-Fe_2_O_3_-modified zeolite composites containing 4, 8, and 12 wt% Fe_2_O_3_ were synthesized and combined with Ca_3_(PO_4_)_2_ to stabilize PTEs in real MSWI bottom ash. Structural and textural properties were examined using XRD, FTIR, BET, SEM, and elemental mapping, while PTE fractionation was evaluated by Tessier sequential extraction and multivariate analysis. Among the investigated loadings, 8 wt% Fe_2_O_3_ provided the best overall stabilization performance within the tested range, reducing the combined labile fractions (F1 + F2) of Cd, Cr, As, and Pb from 41, 42, 35, and 22% to 21, 26, 27, and 13%, respectively. Increasing the loading to 12 wt% produced no further stabilization benefit and was associated with lower BET-derived surface area and pore volume. The observed stabilization performance followed the sequence Pb > Cd > As > Cr > Zn > Cu, accompanied by redistribution from exchangeable and carbonate-bound fractions towards Fe–Mn oxide-associated and residual fractions. Batch-leaching tests further showed 47.8–74.3% and 46.2–68.6% reductions in leachable PTEs under deionized-water and simulated acid-rain conditions, respectively. Collectively, the structural, fractionation, and leaching results are consistent with contributions from zeolite-mediated ion exchange, Fe-associated surface interactions, phosphate-associated processes, and solid-phase evolution during four-week curing. The results show that increasing Fe_2_O_3_ loading alone does not necessarily improve stabilization, emphasizing the need to consider amendment structure, PTE fractionation, and leachability jointly. Further component-resolved experiments, standardized regulatory leaching tests, and long-term ageing studies are required to quantify individual contributions, assess stabilization persistence, and evaluate broader applicability.

## Abstract 2

PTE mobility in MSWI bottom ash is strongly influenced by solid-phase partitioning, not total concentrations alone.

## Graphical abstract

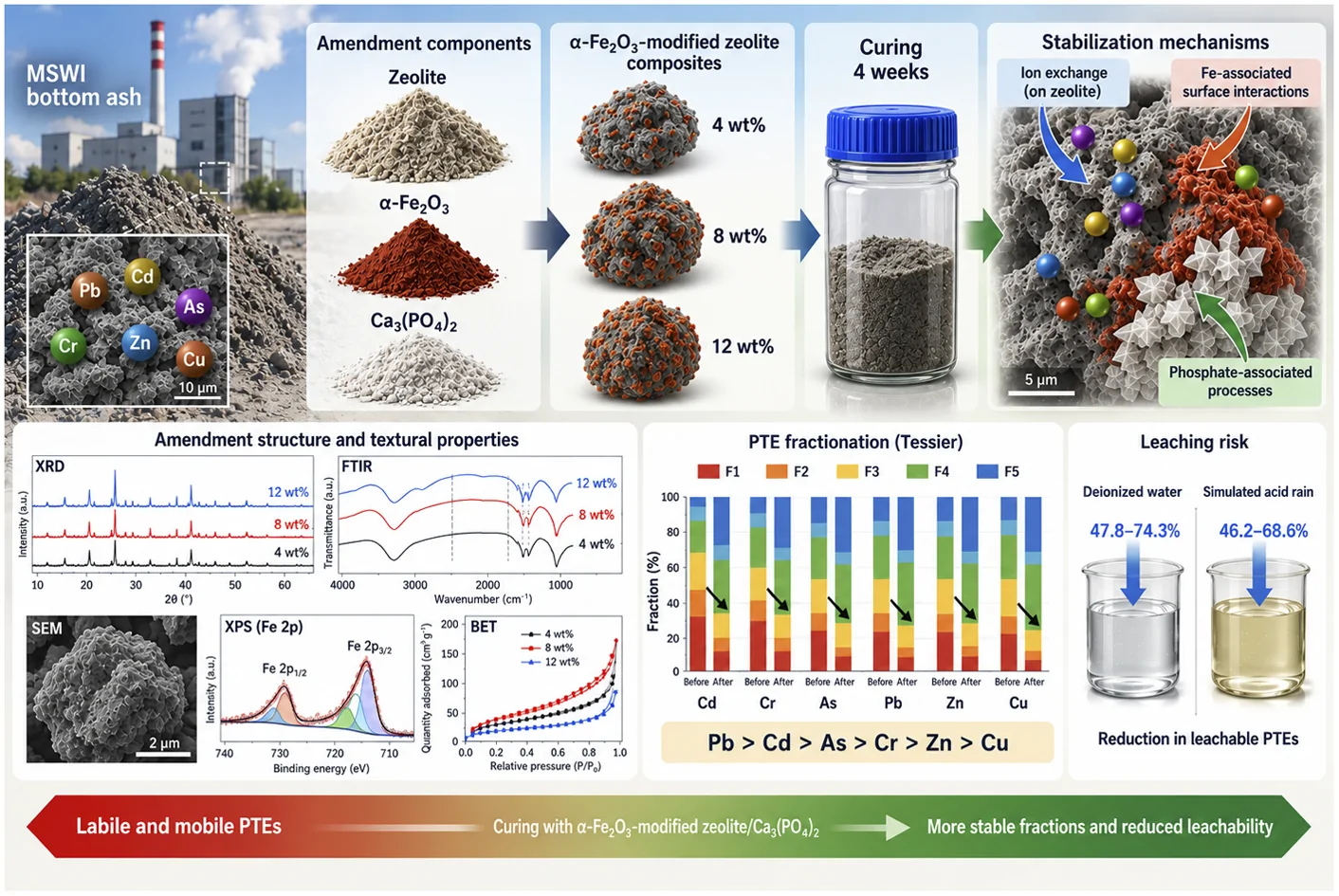

## 1. Introduction

Municipal solid waste incineration (MSWI) is widely adopted because it substantially reduces waste volume while enabling energy recovery and decreasing reliance on landfilling. These environmental benefits are accompanied by the continuous generation of secondary residues, particularly fly ash and bottom ash, which commonly contain elevated concentrations of potentially toxic elements (PTEs), including Pb, Cd, Cr, As, Zn and Cu.^1,2^ During storage, disposal or beneficial reuse, these elements may be gradually released, leading to long-term contamination of surrounding soils and aquatic environments and increasing ecological and human-health risks through bioaccumulation.^3,4^

Assessing the environmental risk of MSWI bottom ash therefore requires consideration of chemical speciation rather than total metal concentrations alone. Metals with comparable bulk concentrations may exhibit markedly different environmental behaviours depending on their distribution among operationally defined fractions. Exchangeable and carbonate-bound species remain highly susceptible to changes in pH, redox conditions and water infiltration, whereas oxide-associated and residual fractions are substantially less mobile and bioavailable.^5–7^ Consequently, long-term stabilization depends not simply on retaining PTEs within the solid matrix but on progressively transforming environmentally labile species into geochemically stable forms. Chemical-speciation analysis therefore provides a more informative assessment of stabilization performance than total metal concentrations or conventional leaching tests alone.^8^

Stabilization/immobilization has consequently become one of the most practical strategies for managing PTE-contaminated solid wastes because it reduces environmental risk without requiring complete metal removal.^9^ Its effectiveness relies on coupled physicochemical processes, including ion exchange, surface complexation, precipitation and mineralization, that progressively redistribute PTEs from mobile fractions (F1–F2) toward increasingly stable fractions (F3–F5), thereby suppressing long-term mobility and bioavailability.^5,8^

Natural zeolite has been extensively investigated as a stabilization material owing to its high cation-exchange capacity, structural stability and abundant exchangeable cations.^10–12^ Nevertheless, pristine zeolite often provides insufficient long-term immobilization in chemically heterogeneous systems such as MSWI bottom ash, where multiple PTEs compete for reactive sites while mineral phases continue to evolve during curing.^13,14^ Surface modification with iron oxides has therefore attracted considerable attention. Among the available iron phases, α-Fe_2_O_3_ is particularly attractive because of its high chemical stability, environmental compatibility, abundant surface hydroxyl groups and strong affinity for metal species.^15–17^ When uniformly dispersed on the zeolite surface, α-Fe_2_O_3_ introduces reactive Fe–OH groups that promote inner-sphere Fe–O–M surface complexation while preserving the intrinsic ion-exchange capability of the aluminosilicate framework.^11,15^ When dispersed on the zeolite surface, α-Fe_2_O_3_ introduces additional Fe-containing interfacial environments that may provide retention sites while preserving the intrinsic ion-exchange capability of the aluminosilicate framework. Phosphate amendments may further contribute to stabilization through interactions with multivalent PTEs and the formation of less labile solid-phase associations. These processes may therefore contribute concurrently to PTE retention during curing, although their relative contributions depend on amendment composition and the evolving geochemical environment.^11,15^

Phosphate amendments can further enhance stabilization by promoting the precipitation of sparingly soluble phosphate minerals, particularly for Pb and other multivalent metals.^18,19^ Within Fe_2_O_3_-modified zeolite systems, phosphate-associated processes may complement zeolite-mediated ion exchange and Fe-associated surface interactions, thereby providing multiple potential retention pathways during curing.^4,5^ Such interactions are expected to be particularly important in real MSWI bottom ash, where multiple PTEs coexist, compete for reactive sites and undergo continuous geochemical redistribution during curing.^2,5^

Despite substantial progress in developing Fe-modified zeolite composites, the mechanisms governing long-term stabilization of PTEs in MSWI bottom ash remain incompletely understood. Most previous studies have emphasized reductions in metal mobility or leachability while providing limited mechanistic insight into how durable immobilization develops throughout curing.^4,11^

Despite these advances, three key mechanistic questions remain unresolved. The first concerns chemical speciation. Stabilization performance has been evaluated predominantly using total metal concentrations or conventional leaching tests, whereas the redistribution of PTEs among operationally defined geochemical fractions has received considerably less attention despite its central importance for long-term environmental stability.^5,6,20^ The second concerns how Fe_2_O_3_ loading influences material evolution and whether these structural changes are associated with subsequent chemical-speciation transformation during stabilization.^11,14^ The third concerns curing dynamics, as stabilization is inherently time dependent yet is commonly evaluated only immediately after treatment, leaving the coupled evolution of material structure and PTE speciation during curing largely unresolved, particularly in chemically heterogeneous MSWI bottom ash.^2,4^

These limitations indicate that a mechanistic framework linking material evolution with chemical-speciation transformation remains lacking. Without such understanding, optimization of stabilization materials continues to rely largely on empirical observations, limiting confidence in predicting their long-term environmental performance. Addressing these questions requires an integrated framework capable of resolving structural evolution, chemical-speciation transformation and curing dynamics as coupled rather than independent processes.

To address these questions, this study combines α-Fe_2_O_3_-modified zeolite with phosphate-assisted stabilization for the treatment of MSWI bottom ash. Rather than evaluating immobilization efficiency alone, the study examines how Fe_2_O_3_ loading is associated with structural evolution, how these structural changes relate to PTE fractionation during curing, and how these coupled changes influence stabilization over the investigated four-week period. To achieve this, α-Fe_2_O_3_modified zeolite composites containing 4, 8 and 12 wt% Fe_2_O_3_ were synthesized and evaluated using real MSWI bottom ash. Structural characterization, Tessier sequential extraction coupled with ICP-OES, hierarchical cluster analysis and principal component analysis were integrated to monitor the coupled evolution of material structure and PTE speciation throughout a four-week curing period.

By linking Fe_2_O_3_ loading, structural evolution, and time-dependent PTE fractionation, this study develops a structure–fractionation framework for interpreting stabilization during the investigated curing period and provides mechanistic insights into the design of zeolite-based amendments for MSWI bottom ash.

## 2. Materials and methods

### 2.1. Preparation of α-Fe_2_O_3_-modified zeolite composites

Unless otherwise specified, all chemicals were of analytical grade and used without further purification. Ferric chloride hexahydrate (FeCl_3_·6H_2_O, ≥99%), sodium hydroxide (NaOH, ≥98%), and calcium phosphate (Ca_3_(PO_4_)_2_, ≥98%) were purchased from Sigma-Aldrich (St. Louis, MO, USA). Deionized (DI) water was used throughout the study.

Natural zeolite served as the support material for preparing the α-Fe_2_O_3_-modified composites. Before synthesis, the zeolite was thoroughly washed with deionized water, dried at 45 °C, and stored in a desiccator. Its mineralogical composition and physicochemical properties are summarized in Table S1 in SI.

For composite preparation, 10.0 g of natural zeolite was dispersed in 100 mL of deionized water and stirred for 15 min to obtain a homogeneous suspension. Subsequently, 100 mL of FeCl_3_·6H_2_O solution containing 1.35, 2.71, or 4.06 g of precursor was slowly introduced under continuous stirring and allowed to react for 40 min. These precursor concentrations corresponded to nominal α-Fe_2_O_3_ loadings of 4, 8, and 12 wt%, expressed as the mass percentage of Fe_2_O_3_ relative to the total composite.

A 2.5 mol per L NaOH solution (approximately 50 mL) was then added dropwise while stirring was maintained for a further 30 min to promote the *in situ* precipitation of iron hydroxide species on the zeolite surface. After precipitation, the suspension was aged for 2 h before being washed repeatedly with deionized water until the filtrate reached approximately neutral pH. The solid product was subsequently air-dried at room temperature (25 ± 2 °C) for 2 h to convert the iron hydroxide precursor into crystalline α-Fe_2_O_3_. Three composite materials were designated as ZF-4, ZF-8, and ZF-12, corresponding to nominal α-Fe_2_O_3_ loadings of 4, 8, and 12 wt%, respectively.

A schematic illustration of the synthesis procedure is shown in Fig. 1.

**Fig. 1:**
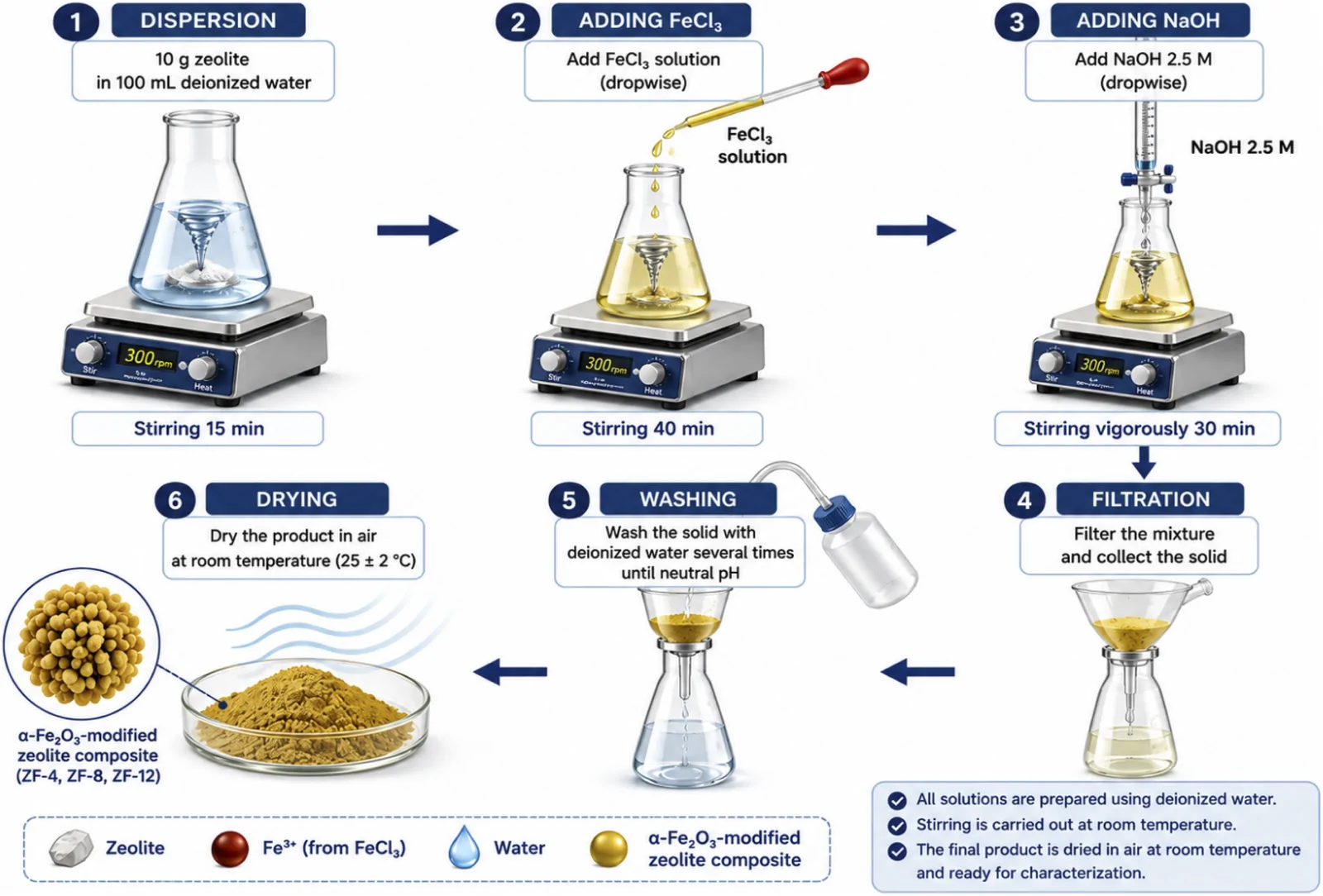
Schematic illustration of the synthesis of α-Fe_2_O_3_-modified zeolite composites (ZF-4, ZF-8, and ZF-12) with nominal Fe_2_O_3_ loadings of 4–12 wt%.

### 2.2. Bottom ash sampling and incubation experiments

#### 2.2.1. Bottom ash collection

Municipal solid waste incineration (MSWI) bottom ash was collected from two municipal solid waste incineration facilities located in Hai Phong and Hanoi, Vietnam, between June and September 2023. Sampling was carried out directly at the bottom-ash discharge points following the Vietnamese standard procedure for solid waste sampling.^21^

Following transportation to the laboratory, the samples were air-dried, homogenized, and sieved through a 2 mm stainless-steel sieve to remove coarse debris. Equal amounts from both facilities were thoroughly blended to obtain a representative composite sample, which was stored in sealed polyethylene containers at room temperature until use.

#### 2.2.2. Effect of α-Fe_2_O_3_ loading on PTE immobilization

The influence of α-Fe_2_O_3_ loading on the immobilization of potentially toxic elements (PTEs) was evaluated using natural zeolite together with α-Fe_2_O_3_-modified zeolite composites containing 4, 8, and 12 wt% Fe_2_O_3_.

Bottom ash (2.0 g), zeolite or α-Fe_2_O_3_-modified zeolite composite (0.50 g), and Ca_3_(PO_4_)_2_ (0.20 g) were thoroughly mixed to obtain a homogeneous solid mixture. The dosages of the stabilization materials were selected based on preliminary optimization experiments to provide sufficient reactive sites while maintaining a practical material-to-ash ratio.

The moisture content was then adjusted with deionized water, after which the beakers were loosely covered with filter paper to minimize evaporation while allowing gas exchange.

Incubation was carried out under laboratory conditions at 28 ± 2 °C. Moisture was inspected every 48 h and replenished with deionized water whenever necessary to maintain approximately 70% of the water-holding capacity throughout the experiment.

#### 2.2.3. Effect of curing time

Following identification of the best-performing α-Fe_2_O_3_ loading within the investigated range, curing experiments were conducted to investigate the temporal evolution of PTE immobilization.

Experimental mixtures were prepared using the procedure described above and incubated for 1, 2, 3, and 4 weeks, respectively.

Untreated bottom ash was incubated under identical conditions and served as the control. At each curing interval, the samples were recovered and dried at 45 °C until constant weight prior to sequential extraction analysis for evaluating changes in PTE fractionation among operationally defined F1–F5 fractions during curing.

### 2.3. Sequential extraction and ICP-OES analysis

Operationally defined PTE fractionations of PTEs were determined using the Tessier sequential extraction procedure,^22^ which separates metals into five operationally defined fractions: F1 (exchangeable), F2 (carbonate-bound), F3 (Fe–Mn oxide-bound), F4 (organic matter-bound), and F5 (residual).

Metal concentrations in each extract were measured using an inductively coupled plasma optical emission spectrometer (ICP-OES, Horiba Ultima 2, Horiba Scientific, Japan) following USEPA Method 6010D.^21,23^

Analytical accuracy was verified using the certified reference material MESS-4, giving recoveries between 90% and 110% (see Table S2 (SI)). Instrument calibration was performed with multi-element standard solutions, while procedural blanks and duplicate analyses were included as part of the quality assurance/quality control (QA/QC) protocol. Detailed operating conditions, analytical wavelengths, limits of detection (LOD), and limits of quantification (LOQ) are summarized in Table S3 (SI).

### 2.4. Material characterization

The crystalline phases of the synthesized composites before and after incubation were identified by X-ray diffraction (XRD, D2 Phaser, Bruker, Germany) using Cu Kα radiation over a 2*θ* range of 20–80°.

Surface functional groups were examined using Fourier-transform infrared spectroscopy (FTIR, JASCO FT/IR-4600, Japan) within the spectral range of 4000–400 cm^−1^.

Morphological features were characterized using field-emission scanning electron microscopy (FE-SEM, JEOL JSM-6700F, Japan), coupled with an Oxford Instruments INCA energy-dispersive X-ray spectroscopy (EDS) detector for elemental analysis and elemental mapping. For semi-quantitative elemental analysis, EDS spectra were acquired from three randomly selected, spatially separated analytical areas on each sample. The resulting elemental compositions are reported as mean ± standard deviation (*n* = 3). Specific surface area, pore volume, and pore-size distribution were obtained from N_2_ adsorption–desorption isotherms (TriStar II 3020, Micromeritics, USA) using the Brunauer–Emmett–Teller (BET) method. Before measurement, the samples were degassed under vacuum following the manufacturer's recommended protocol.

### 2.5. Batch leaching assessment

The leachability of potentially toxic elements (PTEs), including As, Cr, Cd, Pb, Zn, and Cu, was evaluated by comparing untreated and stabilized bottom ash using a batch leaching procedure under deionized-water (DI) and simulated acid-rain (AR) conditions. Untreated bottom ash (T1) and bottom ash stabilized with zeolite/8 wt% α-Fe_2_O_3_/Ca_3_(PO_4_)_2_ for four weeks (T2) were examined, with three independent replicates for each condition (*n* = 3). The 8 wt% Fe_2_O_3_ formulation was selected for the leaching assessment because it provided the best stabilization performance within the investigated loading range.

Samples were dried at 40–45 °C to constant mass, gently ground, and homogenized. A portion of 1.000 ± 0.005 g was transferred into a polypropylene or high-density polyethylene extraction tube, followed by 20.0 mL of extraction solution, corresponding to a liquid-to-solid ratio of 20 mL g^−1^. Two extraction media were employed: DI water and a simulated acid-rain solution. The latter was prepared using H_2_SO_4_/HNO_3_ at a 60 : 40 (w/w) ratio and adjusted to pH 4.20 ± 0.05, based on the acid composition and target pH described for the synthetic precipitation leaching procedure (SPLP; US EPA Method 1312).

The extraction tubes were sealed and agitated at 150 rpm for 24 h at 25 ± 2 °C. Following extraction, the suspensions were centrifuged at 5000 rpm for 10 min, and the supernatants were filtered through 0.45 µm membrane filters. The pH and electrical conductivity (EC) of the filtrates were measured. A 10.0 mL aliquot of each filtrate was acidified with HNO_3_ to pH < 2 and analyzed for As, Cr, Cd, Pb, Zn, and Cu by ICP-OES. Leachable PTE concentrations were expressed as mg L^−1^ and converted to mg kg^−1^ on a dry-mass basis where appropriate.

The apparent immobilization efficiency (*I*_app_) was calculated from the relative reduction in leachable PTE concentration in the stabilized sample relative to untreated bottom ash under the same extraction conditions:1
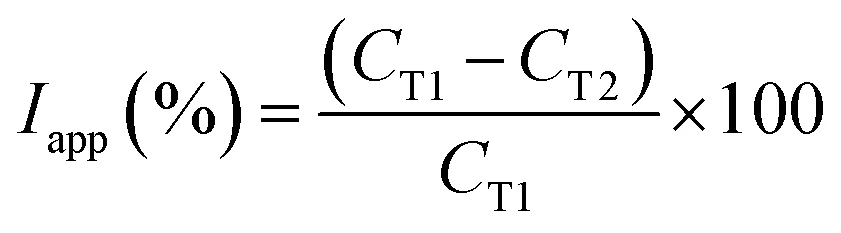
where *C*_T1_ and *C*_T2_ represent the leachable PTE concentrations released from untreated and stabilized bottom ash, respectively, under the same extraction conditions. The same liquid-to-solid ratio and extraction procedure were applied to T1 and T2. No separate correction was applied for the dilution associated with amendment addition; therefore, the calculated values are reported as apparent immobilization efficiencies rather than amendment-mass-corrected immobilization efficiencies. Statistical differences between T1 and T2 were evaluated separately for each extraction medium using one-way analysis of variance (ANOVA), with *p* < 0.05 considered statistically significant.

### 2.6. Statistical analysis

Multivariate statistical analysis was performed to examine relationships among the potentially toxic elements (PTEs; As, Cr, Cd, Pb, Zn, and Cu) and their partitioning among the operationally defined chemical fractions (F1–F5). Prior to PCA and HCA, the data were standardized using *Z*-score transformation to minimize the influence of differences in scale and variance among variables. Principal component analysis (PCA) was used to identify major patterns in PTE fractionation, with the first two principal components (PC1 and PC2) used for graphical representation. Interpretation was based on the explained variance, score distributions, and loading vectors. In the PC1–PC2 space, loading vectors pointing in similar directions were interpreted as positive associations, whereas opposing and approximately orthogonal vectors were interpreted as negative and weak associations, respectively.

Hierarchical cluster analysis (HCA) was performed to assess similarities in the distribution of F1–F5 among untreated and amended bottom-ash samples (M0–M4). Euclidean distance was used as the dissimilarity measure, and Ward's linkage method was applied for hierarchical clustering. The resulting clusters were visualized as a dendrogram. All experiments were conducted in triplicate (*n* = 3), and the results are reported as mean ± standard deviation. Differences among treatments were evaluated using one-way analysis of variance (ANOVA), with *p* < 0.05 considered statistically significant. Statistical analyses and graphical outputs were generated using OriginPro 2021 (OriginLab Corporation, Northampton, MA, USA).

## 3. Results and discussion

### 3.1 Physicochemical characterization of the studied materials

#### 3.1.1. XRD analysis

The X-ray diffraction (XRD) patterns of natural zeolite together with the α-Fe_2_O_3_-modified zeolite composites containing nominal Fe_2_O_3_ loadings of 4, 8, and 12 wt% are presented in Fig. 2.

**Fig. 2:**
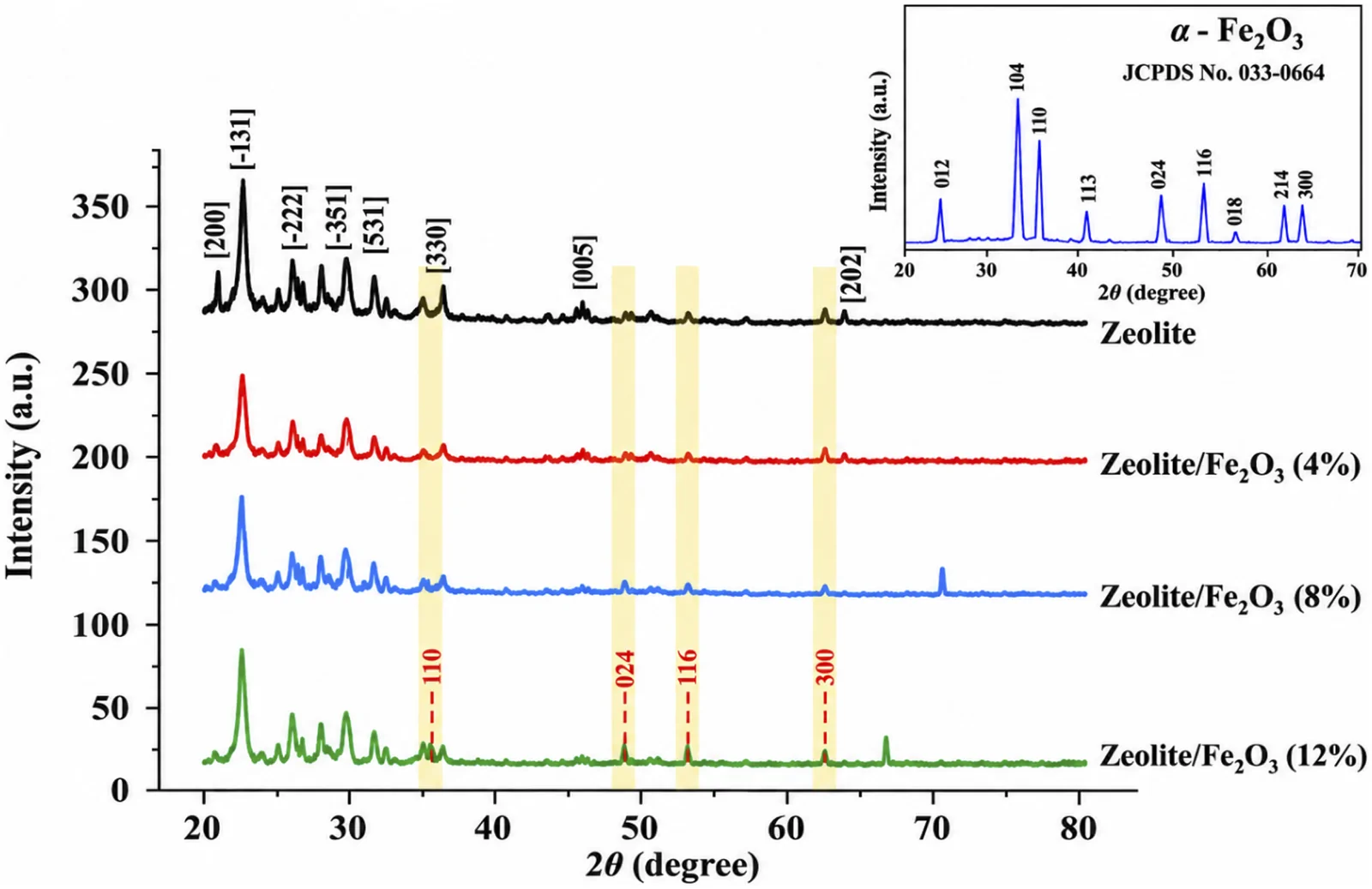
XRD patterns of natural zeolite, α-Fe_2_O_3_-modified zeolite composites containing 4, 8, and 12 wt% Fe_2_O_3_, together with the reference diffraction pattern of α-Fe_2_O_3_.^24^

Natural zeolite exhibited the characteristic diffraction pattern of clinoptilolite, with reflections at 2*θ* = 20.6°, 22.3°, 26.0°, 28.0°, 29.8°, 31.9°, 35.9°, 47.6°, 61.8° and 67.3°, corresponding to the crystallographic planes of the clinoptilolite framework. The reference α-Fe_2_O_3_ pattern was used to identify the deposited iron oxide phase.^24^

Despite the emergence of hematite reflections, the principal clinoptilolite peaks remained essentially unchanged in both position and relative intensity, indicating that the zeolite framework was preserved during surface modification. The coexistence of clinoptilolite and α-Fe_2_O_3_ reflections therefore provides crystallographic evidence for successful incorporation of crystalline hematite without detectable structural alteration of the parent zeolite.^25^

Compared with the reference α-Fe_2_O_3_ pattern, the hematite reflections remained relatively weak, suggesting that Fe_2_O_3_ existed predominantly as a supported phase rather than bulk crystalline hematite, although XRD alone cannot resolve its spatial distribution. The XRD results indicate progressive structural evolution with increasing Fe_2_O_3_ loading, characterized by the formation of crystalline hematite while preserving the clinoptilolite framework. This structural evolution provides the basis for interpreting the subsequent changes in pore texture and PTE stabilization.

#### 3.1.2. FTIR analysis

The Fourier-transform infrared (FTIR) spectra of natural zeolite together with the α-Fe_2_O_3_-modified zeolite composites containing 4, 8, and 12 wt% Fe_2_O_3_ are presented in Fig. 3, alongside the reference spectrum of α-Fe_2_O_3_.^24^

**Fig. 3:**
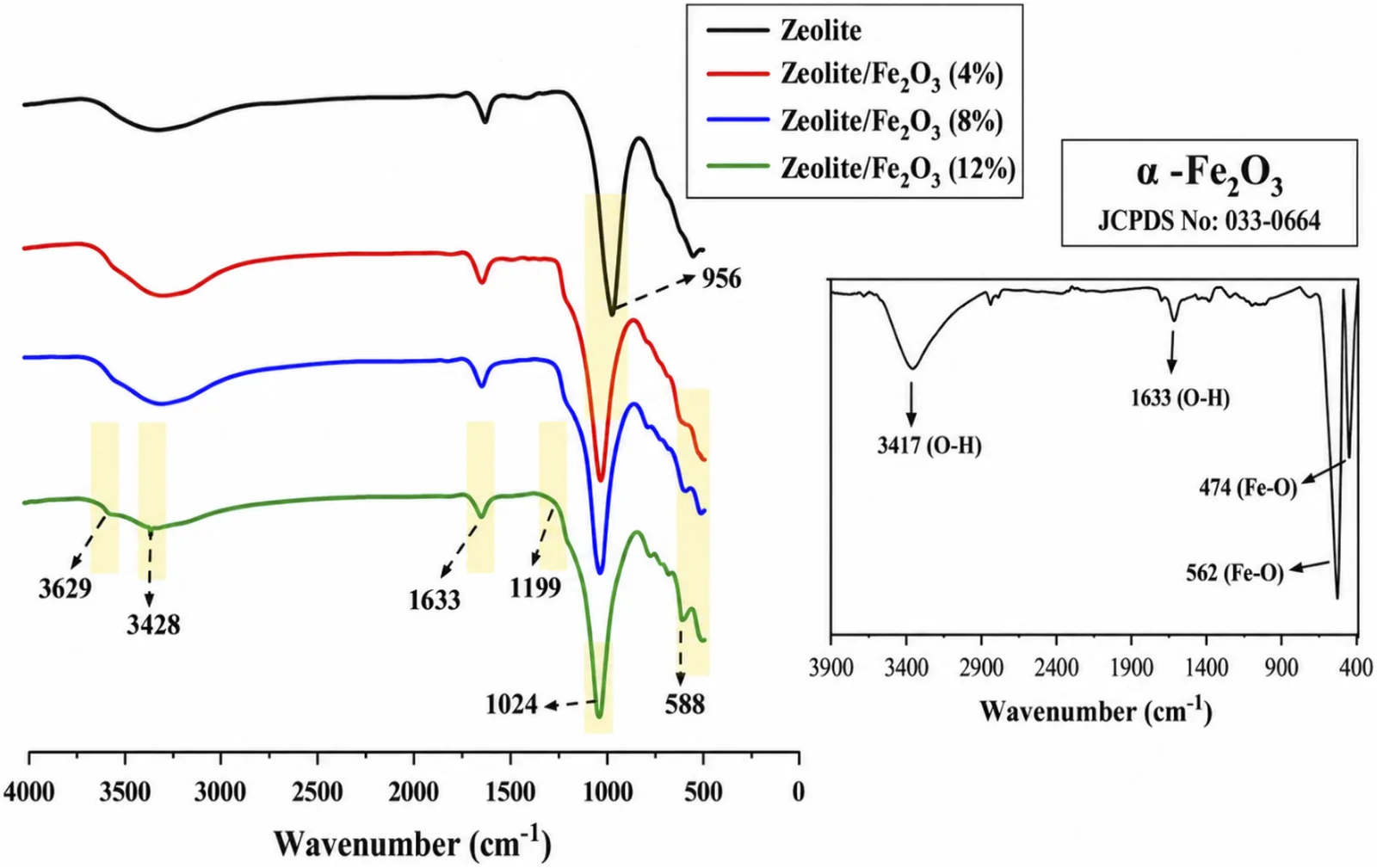
FTIR spectra of natural zeolite and Fe_2_O_3_-modified zeolite composites containing 4, 8, and 12 wt% Fe_2_O_3_, together with the reference spectrum of α-Fe_2_O_3_ (JCPDS No. 33-0664).^24^

A broad band at 3629–3428 cm^−1^, assigned to the stretching vibration of surface hydroxyl groups and hydrogen-bonded water, gradually decreased in intensity following Fe_2_O_3_ deposition. Such attenuation is commonly associated with modification of the surface hydroxyl environment and is consistent with the participation of hydroxyl groups in anchoring deposited iron species.^26^ Since FTIR cannot resolve atomic-scale coordination, this spectral evolution should be regarded as indirect evidence of changes in surface chemistry rather than direct proof of Fe–OH bond formation. A weaker band near 1633 cm^−1^, attributed to the bending vibration of adsorbed water, also changed slightly after modification, indicating an altered local environment for physically adsorbed water.^27^

The characteristic aluminosilicate bands at 1023–1197 cm^−1^ remained well preserved after Fe_2_O_3_ loading. Minor shifts within the 956–1200 cm^−1^ region are commonly associated with localized changes in the Si–O–Al bonding environment caused by surface modification rather than reconstruction of the zeolite framework.^25,27,28^ The preservation of these framework vibrations indicates that α-Fe_2_O_3_ incorporation did not measurably disrupt the structural integrity of the clinoptilolite lattice.

Additional evidence for successful Fe_2_O_3_ incorporation is provided by bands at 461–558 cm^−1^, which correspond to the characteristic Fe–O lattice vibrations of α-Fe_2_O_3_.^24^ These bands provide complementary spectroscopic evidence for hematite formation, in agreement with previous reports and the XRD results.^25,27,29^

#### 3.1.3. BET analysis

The textural properties of natural zeolite and α-Fe_2_O_3_-modified zeolite composites were evaluated by N_2_ adsorption–desorption analysis. The corresponding adsorption–desorption isotherms are presented in Fig. 4, while the resulting textural parameters (Table 1) reveal how Fe_2_O_3_ loading modified the pore structure of the composites.

**Fig. 4:**
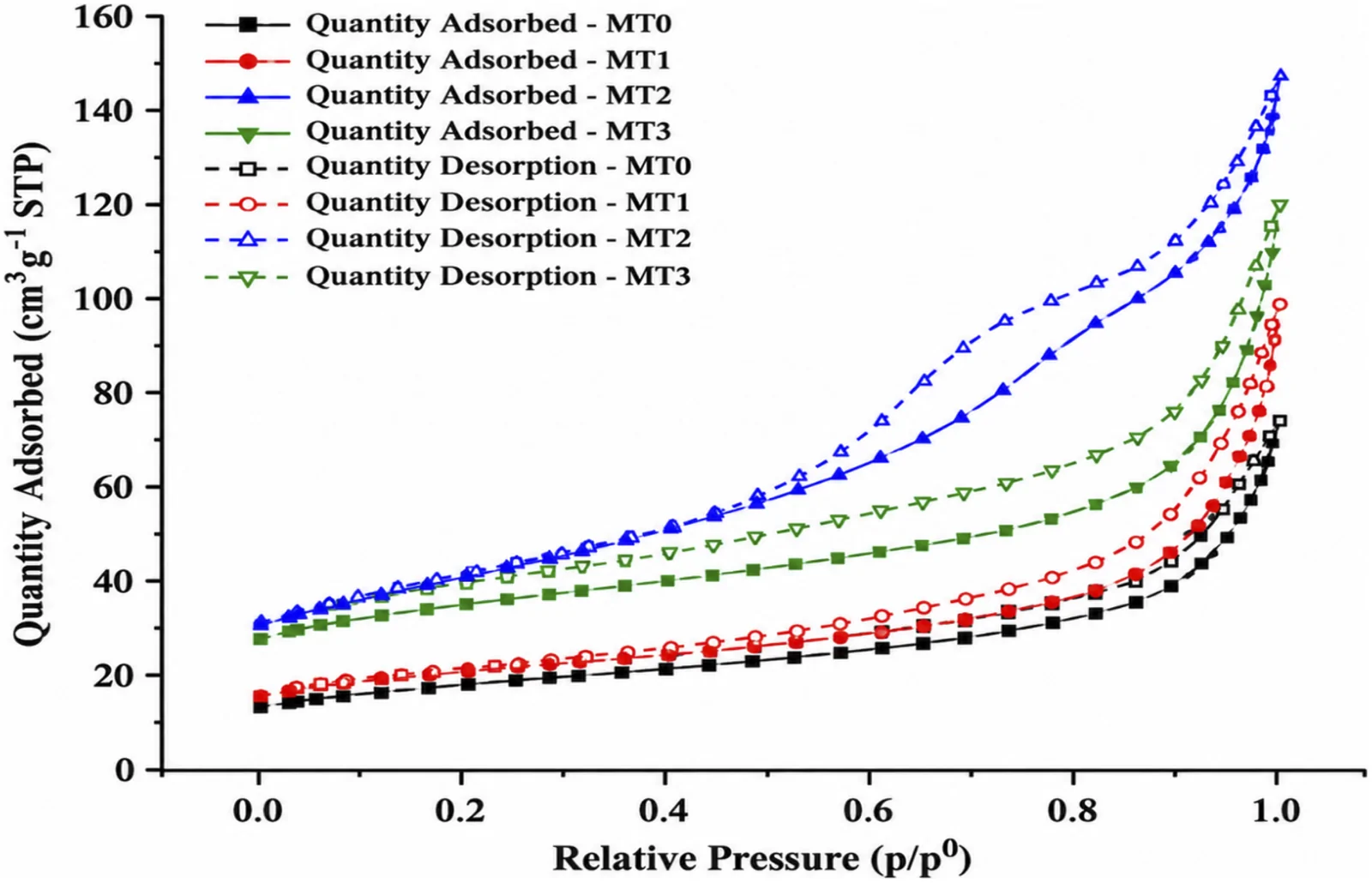
N_2_ adsorption–desorption isotherms of natural zeolite (MT0) and Fe_2_O_3_-modified zeolite composites containing 4 wt% (MT1), 8 wt% (MT2), and 12 wt% Fe_2_O_3_ (MT3).

**Table 1:** BET-derived textural properties of natural zeolite and Fe_2_O_3_-modified zeolite composites

Sample	*S*_BET_ (m^2^ g^−1^)	*V*_pore_ (cm^3^ g^−1^)	*D*_pore_ (nm)
Zeolite – MT0	41.69	0.1016	12.65
Zeolite/Fe_2_O_3_ (4%)	43.86	0.1475	12.85
Zeolite/Fe_2_O_3_ (8%)	114.73	0.239	7.996
Zeolite/Fe_2_O_3_ (12%)	90.60	0.1591	11.43

Pristine zeolite exhibited a relatively low specific surface area (41.69 m^2^ g^−1^), pore volume (0.1016 cm^3^ g^−1^) and an average pore diameter of 12.65 nm. Incorporation of 4 wt% Fe_2_O_3_ produced only minor increases in surface area and pore volume while leaving the average pore diameter essentially unchanged, indicating that limited Fe_2_O_3_ deposition exerted little influence on the pore architecture of the parent zeolite.

A markedly different response occurred at 8 wt% Fe_2_O_3_, where the specific surface area increased to 114.73 m^2^ g^−1^, accompanied by the highest pore volume (0.2390 cm^3^ g^−1^) and the smallest average pore diameter (8.00 nm). Considered together, these changes suggest the development of additional mesoporosity rather than simple pore enlargement, plausibly arising from finely dispersed Fe_2_O_3_ particles that generated interparticle voids accessible to N_2_ molecules. Because BET probes pore texture rather than particle morphology, this interpretation should be regarded as mechanistically consistent rather than direct evidence of nanoparticle dispersion.

Increasing the Fe_2_O_3_ loading to 12 wt% reduced both the specific surface area (90.60 m^2^ g^−1^) and pore volume (0.1591 cm^3^ g^−1^), whereas the average pore diameter increased to 11.43 nm. Together with the SEM observations, these changes are consistent with increased Fe_2_O_3_ deposition and particle aggregation that may partially restrict pore accessibility at the highest loading. Because BET does not directly resolve the location or nature of pore restriction, this interpretation represents a plausible structure–texture relationship rather than direct evidence of internal pore blockage.

According to the IUPAC classification, all samples exhibited Type IV adsorption–desorption isotherms with a pronounced hysteresis loop over *P*/*P*_0_ ≈ 0.4–1.0, characteristic of mesoporous materials. Nitrogen adsorption increased in the order: ZF-8 > ZF-12 > ZF-4 > pristine zeolite, which agrees well with the BET surface area and pore-volume data. The more pronounced hysteresis after Fe_2_O_3_ modification further suggests that Fe_2_O_3_ deposition altered the mesoporous texture of the composites, although the adsorption isotherms alone cannot resolve the detailed pore geometry.

Among the investigated composites, 8 wt% Fe_2_O_3_ exhibited the most favourable textural characteristics, whereas further Fe_2_O_3_ addition reduced the available surface area and pore volume. This trend agrees closely with the XRD and FTIR results, which indicate progressive incorporation of the α-Fe_2_O_3_ phase while preserving the clinoptilolite framework. These complementary characterization results establish a coherent picture of structural evolution, providing the basis for evaluating the stabilization of PTEs.

#### 3.1.4. SEM and EDS analyses

SEM-EDS characterization provided additional evidence for the presence of an iron-containing phase on the zeolite surface (Fig. 5a–f). Elemental compositions were determined from three independent analytical areas for each material and are reported as mean ± standard deviation (*n* = 3). Natural clinoptilolite exhibited the characteristic aluminosilicate morphology, whereas Fe_2_O_3_ modification resulted in a visibly rougher surface morphology while broadly preserving the original particle morphology (Fig. 6A–C).

**Fig. 5:**
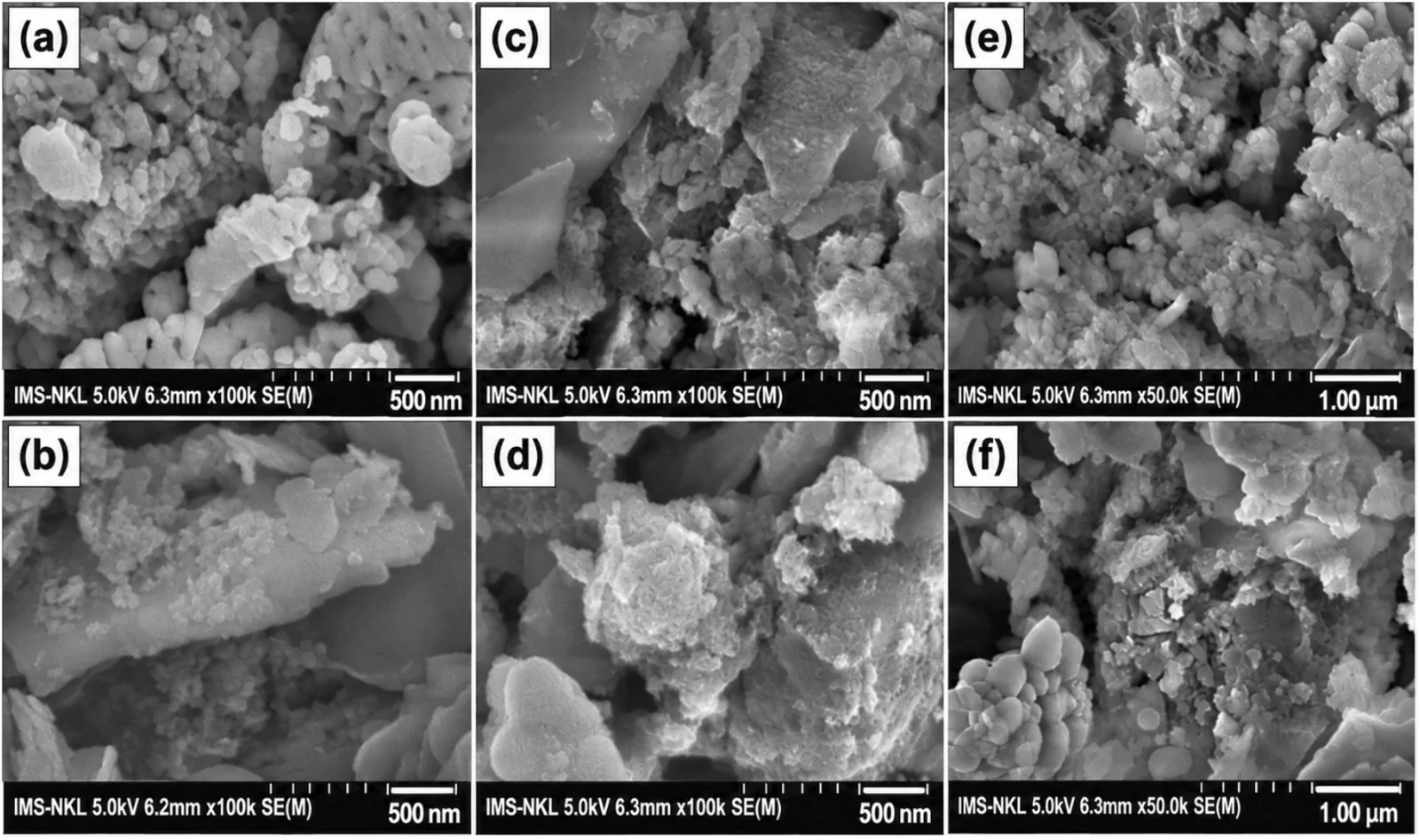
SEM micrographs of the initial zeolite-based materials: (a–d) M1–M4 at 100 000× (500 nm scale bar) and (e and f) M3 and M4 at 50 000× (1 µm scale bar). M1 denotes the natural-zeolite material, whereas M2–M4 contain 4, 8, and 12 wt% Fe_2_O_3_-modified zeolite, respectively.

**Fig. 6:**
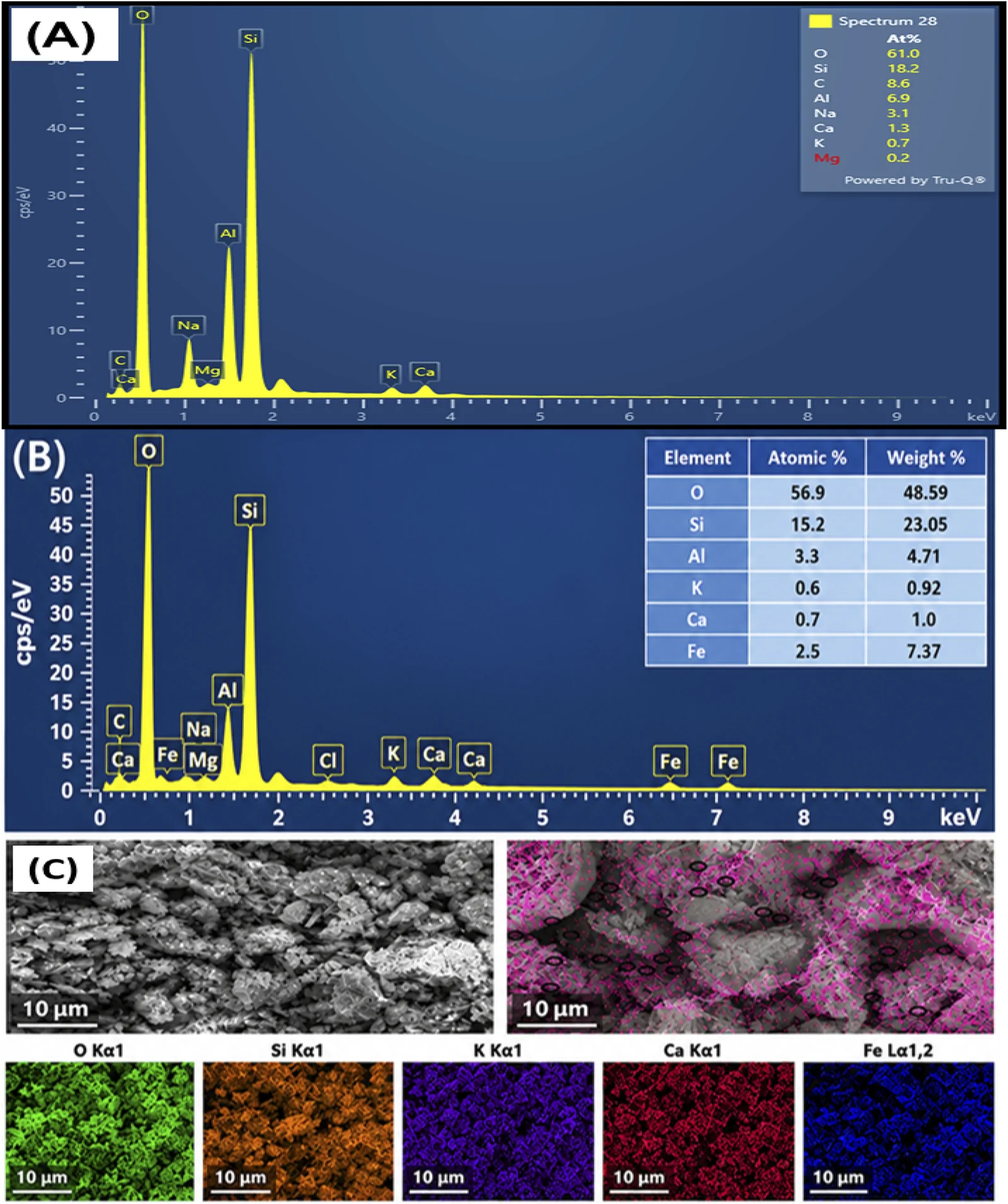
EDS spectra of natural zeolite (A) and the Fe_2_O_3_-modified zeolite composite (B), together with the corresponding elemental mapping images (C) showing the spatial distribution of Fe, O, Si, Ca and Al.

The initial materials exhibited heterogeneous morphologies comprising irregular particles, aggregates, and interparticle voids (Fig. 5a–d). With increasing Fe_2_O_3_ loading, changes in surface texture and particle aggregation became more apparent. M1 exhibited the characteristic morphology of the natural-zeolite material, whereas M2 showed increased surface roughness following incorporation of 4 wt% Fe_2_O_3_. M3 (8 wt% Fe_2_O_3_) retained discernible interparticle spaces, whereas further increasing the loading to 12 wt% produced denser surface regions and more pronounced fine-particle accumulation in M4 (Fig. 5c and d). The corresponding 50 000× images further resolve the difference between M3 and M4, with M4 showing more localized compact regions and particle aggregation (Fig. 5e and f).

Relative to M3, the denser morphology of M4 is consistent with its lower BET surface area and pore volume and may contribute to reduced pore accessibility. This observation suggests that increasing Fe_2_O_3_ loading beyond 8 wt% does not necessarily improve textural accessibility. However, SEM does not directly resolve restriction within the internal pore network; therefore, the observed morphology is interpreted as evidence consistent with reduced pore accessibility rather than direct evidence of pore blockage. Other loading-dependent effects may also contribute to the decline in BET-derived textural parameters.

SEM-EDS detected Fe at 4.4 ± 0.2 at% in the Fe_2_O_3_-modified zeolite, whereas Fe was not detected in the analysed areas of the natural zeolite (Table 2). The modified zeolite also exhibited lower relative abundances of O, Si, and Al, with Si showing the most pronounced decrease from 20.2 ± 3.2 to 6.1 ± 1.3 at%. In contrast, the relative abundances of C, Ca, and Mg increased after modification, whereas K changed only slightly. In particular, the pronounced increase in Ca from 1.2 ± 0.3 to 11.7 ± 2.0 at% indicates that the shift in normalized elemental abundances cannot be attributed to Fe_2_O_3_ deposition alone. Because SEM-EDS provides semi-quantitative information from the analysed region rather than bulk elemental concentrations, these variations are interpreted as changes in the local elemental composition sampled by EDS rather than as changes in the bulk composition of the zeolite framework.

**Table 2:** Semi-quantitative elemental composition (atomic %) of natural zeolite and the Fe_2_O_3_-modified zeolite composite determined by SEM-EDS. Values are presented as mean ± standard deviation from replicate EDS measurements performed on randomly selected analytical areas (*n* = 3)^a^

Element	Zeolite (%)	Zeolite/Fe_2_O_3_ (%)
O	60.0 ± 3.6	51.5 ± 2.1
Si	20.2 ± 3.2	6.1 ± 1.3
C	11.6 ± 5.6	17.2 ± 0.5
Al	4.9 ± 1.3	2.3 ± 0.5
Ca	1.2 ± 0.3	11.7 ± 2.0
K	0.9 ± 0.2	0.7 ± 0.2
Mg	0.4 ± 0.1	2.34 ± 0.2
Fe	ND	4.4 ± 0.2

a ND: not detected in the analysed areas by SEM-EDS.

The decrease in the relative Si and Al abundances may be associated with partial coverage of the analysed zeolite surface by the deposited iron-containing phase, which reduces the relative contribution of the underlying aluminosilicate matrix to the EDS signal. This interpretation is consistent with the XRD and FTIR results, which showed that the characteristic structural features of clinoptilolite were largely retained after modification. Elemental mapping further indicated that Fe was broadly distributed across the analysed zeolite surface rather than confined to localized regions (Fig. 6C). Although EDS mapping cannot resolve nanoscale dispersion or particle size, the observed Fe distribution is consistent with relatively widespread deposition of the iron-containing phase over the analysed zeolite surface. The relatively weak hematite reflections in the XRD patterns are likewise consistent with α-Fe_2_O_3_ being present as a relatively minor crystalline component.

Across the investigated loadings, the morphological evolution was consistent with the variation in BET-derived textural properties. M3 (8 wt% Fe_2_O_3_) exhibited the most favourable combination of textural characteristics among the investigated loadings, whereas the denser surface features and more pronounced aggregation observed for M4 (12 wt% Fe_2_O_3_) provide complementary morphological support for the decrease in BET-derived textural properties at higher loading.

#### 3.1.5. XPS analysis of surface chemical states

X-ray photoelectron spectroscopy (XPS) was employed to further characterize the near-surface chemical environment of the α-Fe_2_O_3_-modified zeolite composite (Fig. 7).

**Fig. 7:**
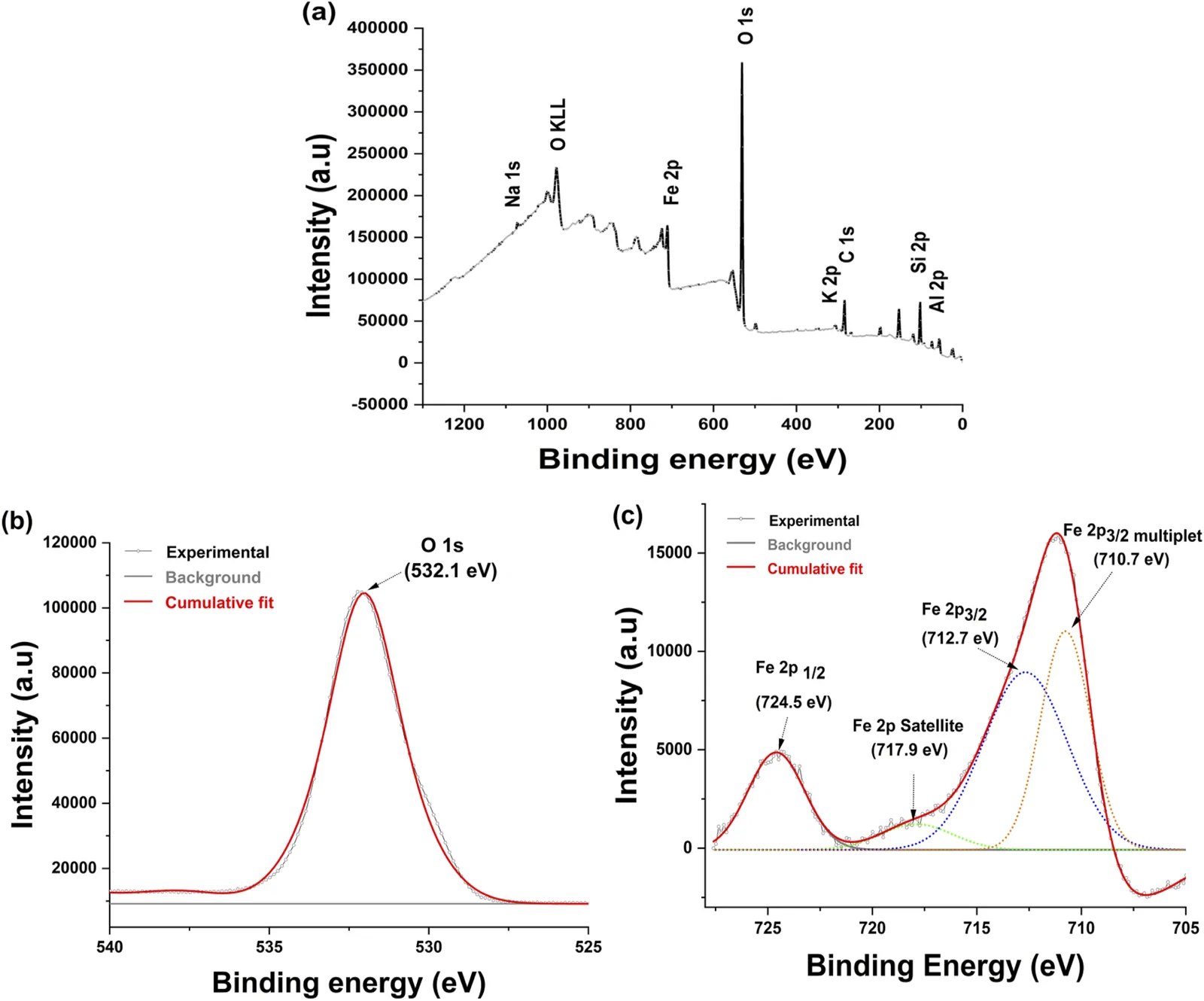
XPS characterization of the α-Fe_2_O_3_-modified zeolite composite: (a) survey spectrum, (b) O 1s spectrum, and (c) Fe 2p spectrum with corresponding fitted components.

The survey spectrum (Fig. 7a) shows characteristic signals associated with O, Si, Al, and Fe, together with minor contributions from other surface-associated elements, providing surface-sensitive evidence for the presence of Fe in the near-surface region of the composite. The Fe signal complements the crystalline-phase identification obtained by XRD and the vibrational information provided by FTIR.

The O 1s spectrum (Fig. 7b) shows a broad envelope centred at approximately 532.1 eV. Given the overlapping contributions from oxygen associated with the aluminosilicate framework, surface hydroxyl groups, iron-containing species, and potentially phosphate-containing environments, the O 1s region is treated here as a composite envelope rather than as individually resolved chemical components. Accordingly, the envelope is interpreted as representing the overall near-surface oxygen environment rather than a uniquely assigned oxygen species. The Fe 2p spectrum (Fig. 7c) exhibits the characteristic Fe 2p spin–orbit doublet, with fitted Fe 2p_3/2_ components at approximately 710.7 and 712.7 eV and the Fe 2p_1/2_ component at 724.5 eV, together with a satellite feature near 717.9 eV. The spectral profile is consistent with an Fe^3+^-rich iron-oxide chemical environment, in agreement with the α-Fe_2_O_3_ phase identified by XRD.

Together, the XPS results provide surface-sensitive chemical-state evidence for the Fe_2_O_3_-modified zeolite and complement the structural information obtained from XRD, FTIR, BET, and SEM-EDS. The data support the presence of Fe-containing surface species and provide additional information on their near-surface chemical environment. These observations establish the initial surface-chemical characteristics of the modified zeolite; their relevance to curing-induced changes and PTE stabilization is considered subsequently in Section 3.5.

### 3.2. Effect of Fe_2_O_3_ loading on PTE speciation and stabilization in municipal solid waste incineration bottom ash

The influence of Fe_2_O_3_ loading (4, 8, and 12 wt%) on the stabilization of PTEs in municipal solid waste incineration bottom ash was systematically investigated. To enhance the immobilization of mobile PTE species, particularly Pb and Cd, which are commonly enriched in bottom ash,^30^ Ca_3_(PO_4_)_2_ was incorporated into the curing system. Structural evolution during curing was subsequently characterized by XRD, FTIR, and SEM-EDS to relate mineral evolution to the observed changes in PTE speciation.

#### 3.2.1. XRD analysis of cured bottom ash

The X-ray diffraction patterns of untreated bottom ash (M0) together with samples cured using natural zeolite (M1) and Fe_2_O_3_-modified zeolite composites containing 4 wt% (M2), 8 wt% (M3), and 12 wt% (M4) Fe_2_O_3_ are presented in Fig. 8. Whereas Section 3.1.1 established the successful incorporation of crystalline α-Fe_2_O_3_ into the zeolite support while preserving the clinoptilolite framework, the present diffraction patterns characterize mineral evolution within the curing matrix. Compared with untreated bottom ash, all cured samples exhibit noticeable variations in peak intensity together with several newly developed reflections, particularly within the regions of approximately 20–35.6°, 39–43°, around 60°, and 64–68° (2*θ*). Rather than representing simple superposition of the starting materials, these features reflect progressive reorganization of the mineral assemblage during curing. The underlying mechanisms cannot be resolved from XRD alone and are therefore interpreted together with the complementary analyses presented below.^2,30^

**Fig. 8:**
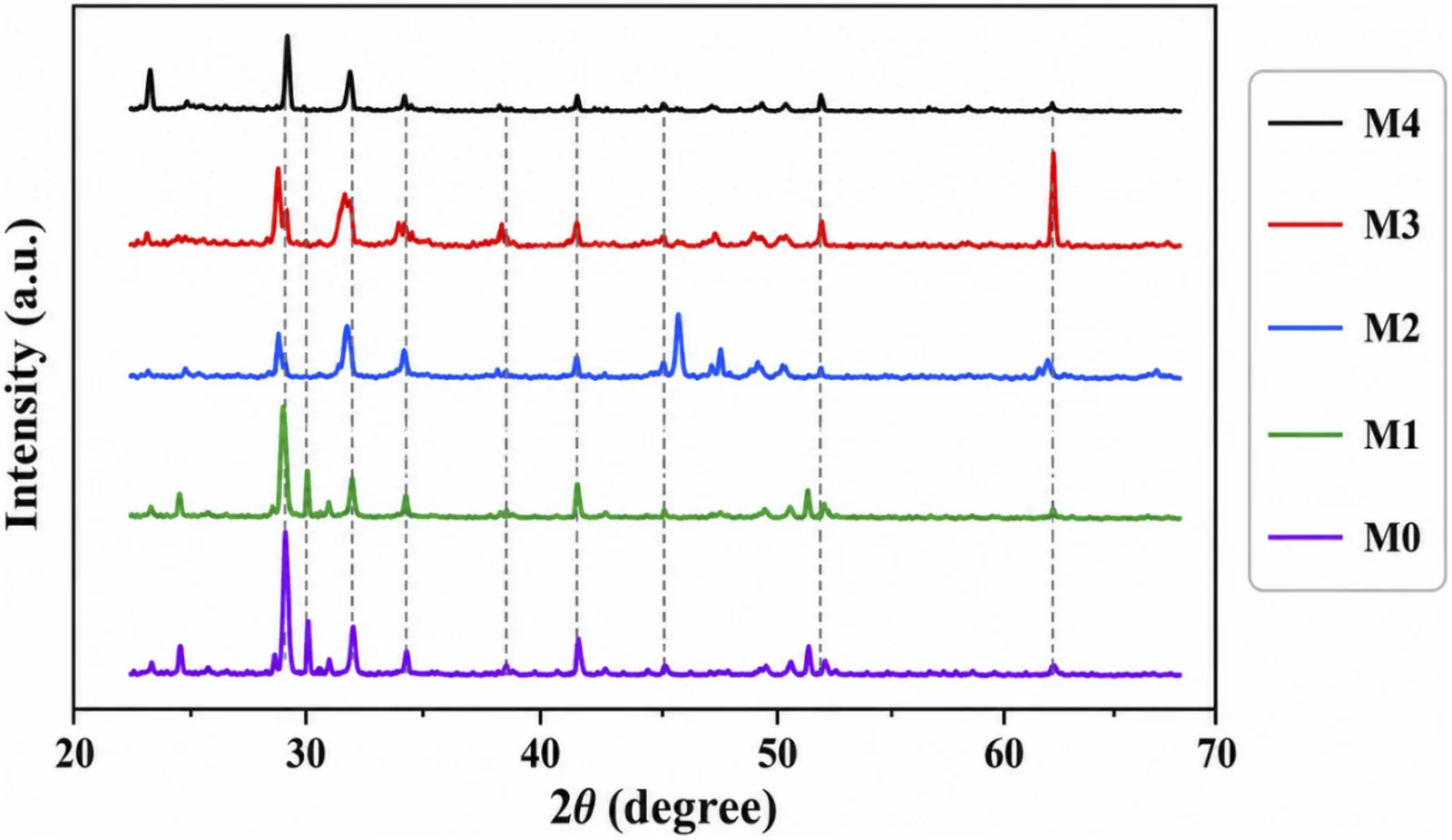
XRD patterns of untreated bottom ash (M0) and bottom ash after curing with natural zeolite (M1) and Fe_2_O_3_-modified zeolite composites containing 4 wt% (M2), 8 wt% (M3), and 12 wt% Fe_2_O_3_ (M4).

Only subtle crystallographic changes are observed after treatment with natural zeolite (M1). Most reflections associated with the original ash matrix remain recognizable, accompanied by several weak additional peaks between approximately 25.6° and 31.6° (2*θ*). The limited extent of these changes suggests relatively modest mineral transformation, with any newly formed phases contributing only weakly to the overall diffraction pattern. Similar behaviour has been reported for natural zeolite systems, where curing induces only limited modification of the existing crystalline assemblage in the absence of additional reactive surface phases.^14^

Introducing Fe_2_O_3_-modified zeolite produces more pronounced crystallographic changes. At 4 wt% Fe_2_O_3_ (M2), several additional reflections become discernible, although their intensity remains relatively low. Increasing the loading to 8 wt% (M3) results in more clearly resolved reflections within approximately 29–31°, 35.7–43°, 49.8°, and 59.5°, together with increased intensity of several existing peaks. Collectively, these observations indicate more extensive crystallization than observed for M2. Increased diffraction intensity should not, however, be interpreted solely as evidence of greater iron content. Variations in crystallinity, relative phase abundance, and preferred crystal orientation may all contribute to the observed pattern. Within the limits of XRD, these observations are consistent with stabilization pathways reported for zeolite- and phosphate-based systems, including ion exchange, surface complexation and precipitation, although confirmation requires complementary analytical evidence.^31^

Further increasing the Fe_2_O_3_ loading to 12 wt% (M4) produced reflections that were sharper and better resolved than those observed for M3. Such behaviour may reflect either improved crystallinity or the development of larger crystalline domains as Fe_2_O_3_ loading increased. XRD alone cannot distinguish between these possibilities. Unlike the synthesized composites discussed in Section 3.1.1, the present diffraction patterns primarily reflect mineral evolution within the curing matrix rather than structural modification of the zeolite support.^32,33^

Overall, XRD demonstrates progressive mineral evolution during curing with Fe_2_O_3_-modified zeolite but cannot by itself resolve the underlying stabilization mechanisms. These are therefore interpreted together with the FTIR, SEM-EDS, and sequential extraction results presented in the following sections.

#### 3.2.2. FTIR analysis of cured bottom ash

Following the XRD analysis presented in Section 3.2.1, Fourier-transform infrared (FTIR) spectroscopy was employed to examine how the chemical environment of the curing system evolved after treatment with natural zeolite (M1) and α-Fe_2_O_3_-modified zeolite containing 4 wt% (M2), 8 wt% (M3), and 12 wt% (M4) Fe_2_O_3_ in the presence of Ca_3_(PO_4_)_2_. The corresponding spectra are presented in Fig. 9. By probing local bonding environments rather than long-range crystal order, FTIR provides a spectroscopic perspective that complements the crystallographic information obtained from XRD. Broad hydroxyl absorption centred within 3400–3600 cm^−1^ gradually decreases in intensity from M1 to M4. Compared with untreated bottom ash (M0), natural zeolite produces only modest attenuation, whereas substantially greater reductions accompany incorporation of the α-Fe_2_O_3_-modified zeolite composites. This progressive change suggests modification of the surrounding hydroxyl environment during curing. FTIR, however, cannot resolve the corresponding coordination chemistry, and these spectral variations should therefore be regarded as indirect evidence rather than proof of specific precipitation reactions or Fe–OH bond formation.^34^ The weaker band located near 1600–1640 cm^−1^, assigned to the bending vibration of molecularly bound water and surface hydroxyl species, also becomes progressively more distinguishable after treatment, implying changes in the hydration environment surrounding the solid matrix.^34,35^

**Fig. 9:**
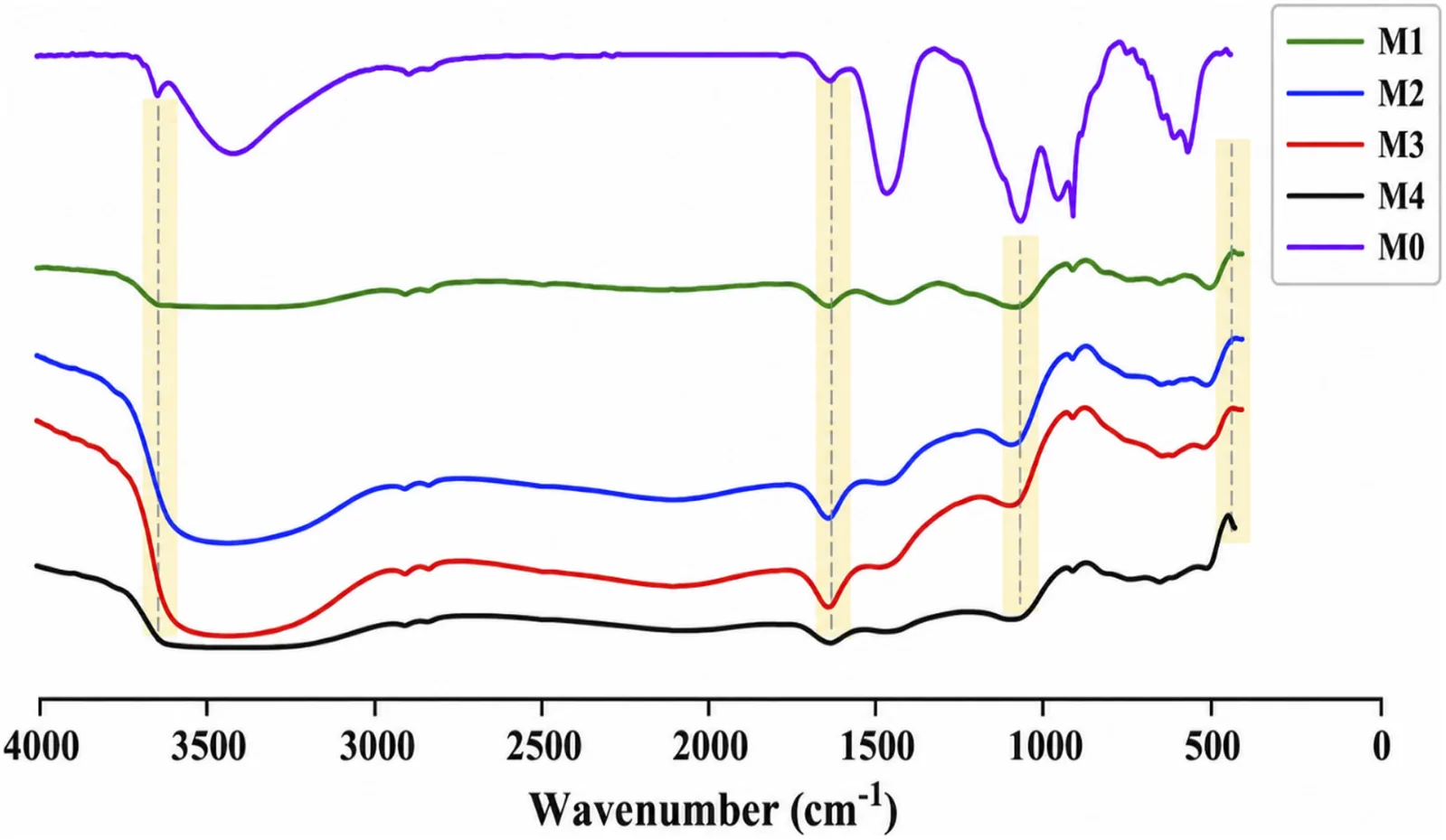
FTIR spectra of untreated municipal solid waste incineration bottom ash (M0) and bottom ash cured with natural zeolite (M1) and Fe_2_O_3_-modified zeolite composites containing 4 wt% (M2), 8 wt% (M3), and 12 wt% (M4) Fe_2_O_3_.

Within the 1000–1100 cm^−1^ region, the principal aluminosilicate vibrations gradually broaden and exhibit slight distortion as the Fe_2_O_3_ loading increases. Rather than indicating reconstruction of the zeolite framework, these subtle spectral variations are more plausibly associated with changes in the surrounding chemical environment of the aluminosilicate network introduced during curing.^36^ This interpretation agrees well with the diffraction data presented in Section 3.2.1, where the principal zeolite framework remained recognizable despite progressive crystallographic evolution. Viewed together, the XRD and FTIR results suggest that curing primarily modifies short-range bonding environments while preserving the fundamental aluminosilicate framework.

Low-wavenumber vibrations between 500 and 600 cm^−1^ become progressively more evident with increasing Fe_2_O_3_ loading. Their positions coincide with the characteristic Fe–O lattice vibrations reported for hematite.^37,38^ Whether these absorptions arise exclusively from the deposited α-Fe_2_O_3_ or also involve iron-bearing environments generated during curing cannot be resolved by FTIR alone. Accordingly, assignment of these bands requires interpretation alongside the XRD and SEM-EDS observations rather than on the basis of infrared spectroscopy alone.

Despite exhibiting the strongest attenuation of the hydroxyl band together with the most pronounced low-wavenumber features, M4 should not be regarded as demonstrating superior stabilization solely on the basis of its FTIR spectrum. These spectral differences may equally reflect a greater contribution of iron-containing surface species or progressive modification of the surrounding coordination environment associated with increasing Fe_2_O_3_ loading.^39^ Their relevance to PTE stabilization is therefore evaluated subsequently using sequential extraction together with multivariate statistical analysis.

The FTIR spectra capture progressive spectroscopic evolution accompanying the curing process. On their own, these data cannot identify the reactions responsible for the observed spectral changes; interpreted together with the XRD, SEM-EDS, and sequential extraction results presented in the following sections, they provide complementary evidence for understanding the structural development of the curing system.

#### 3.2.3. SEM-EDS analyses of cured bottom ash

The morphology and elemental composition of the cured bottom ash were examined by SEM coupled with EDS. Representative SEM micrographs are shown in Fig. 10, while the corresponding EDS spectra and elemental maps are presented in Fig. 11 and 12. These observations provide complementary information on the microstructural evolution of the curing matrix.

**Fig. 10:**
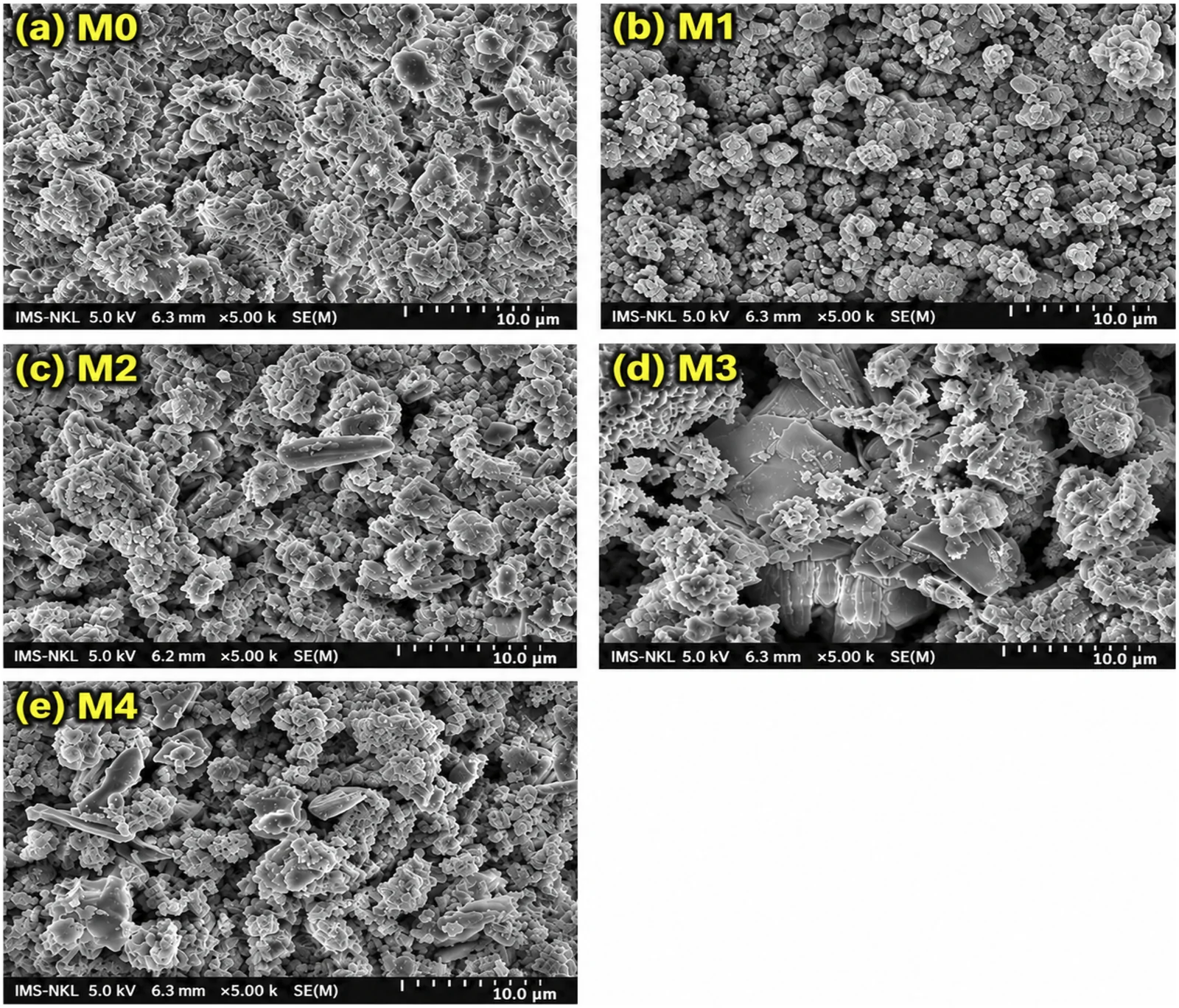
SEM micrographs of (a) untreated bottom ash (M0); (b) bottom ash cured with natural zeolite (M1); and bottom ash cured with Fe_2_O_3_-modified zeolite/Ca_3_(PO_4_)_2_ composites containing (c) 4 wt% (M2), (d) 8 wt% (M3), and (e) 12 wt% (M4) Fe_2_O_3_.

**Fig. 11:**
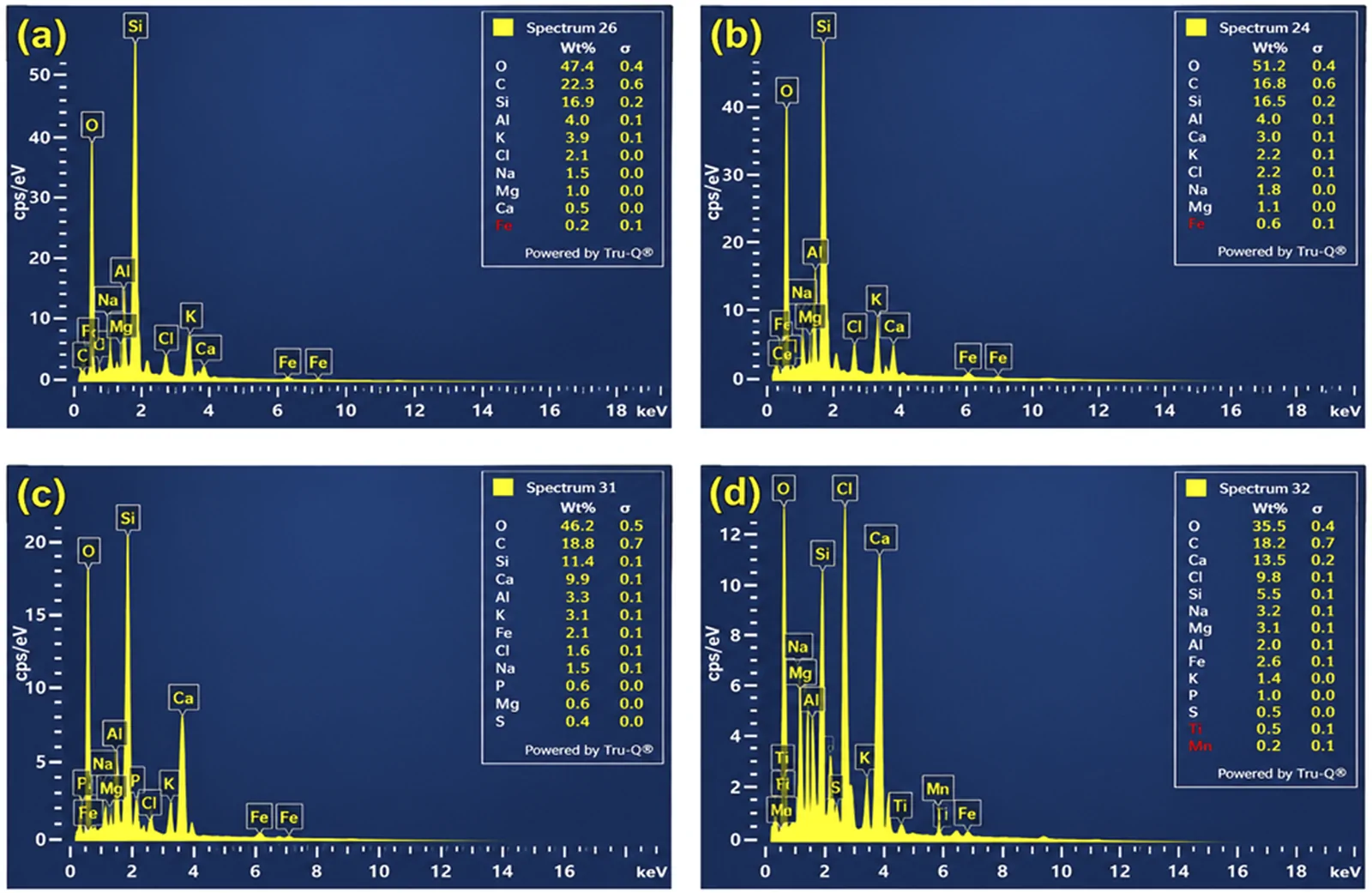
EDS spectra of untreated bottom ash (a), bottom ash cured with natural zeolite (b), Fe_2_O_3_-modified zeolite (8 wt%, (c)), and Fe_2_O_3_-modified zeolite/Ca_3_(PO_4_)_2_ composite (8 wt% Fe_2_O_3_, (d)).

**Fig. 12:**
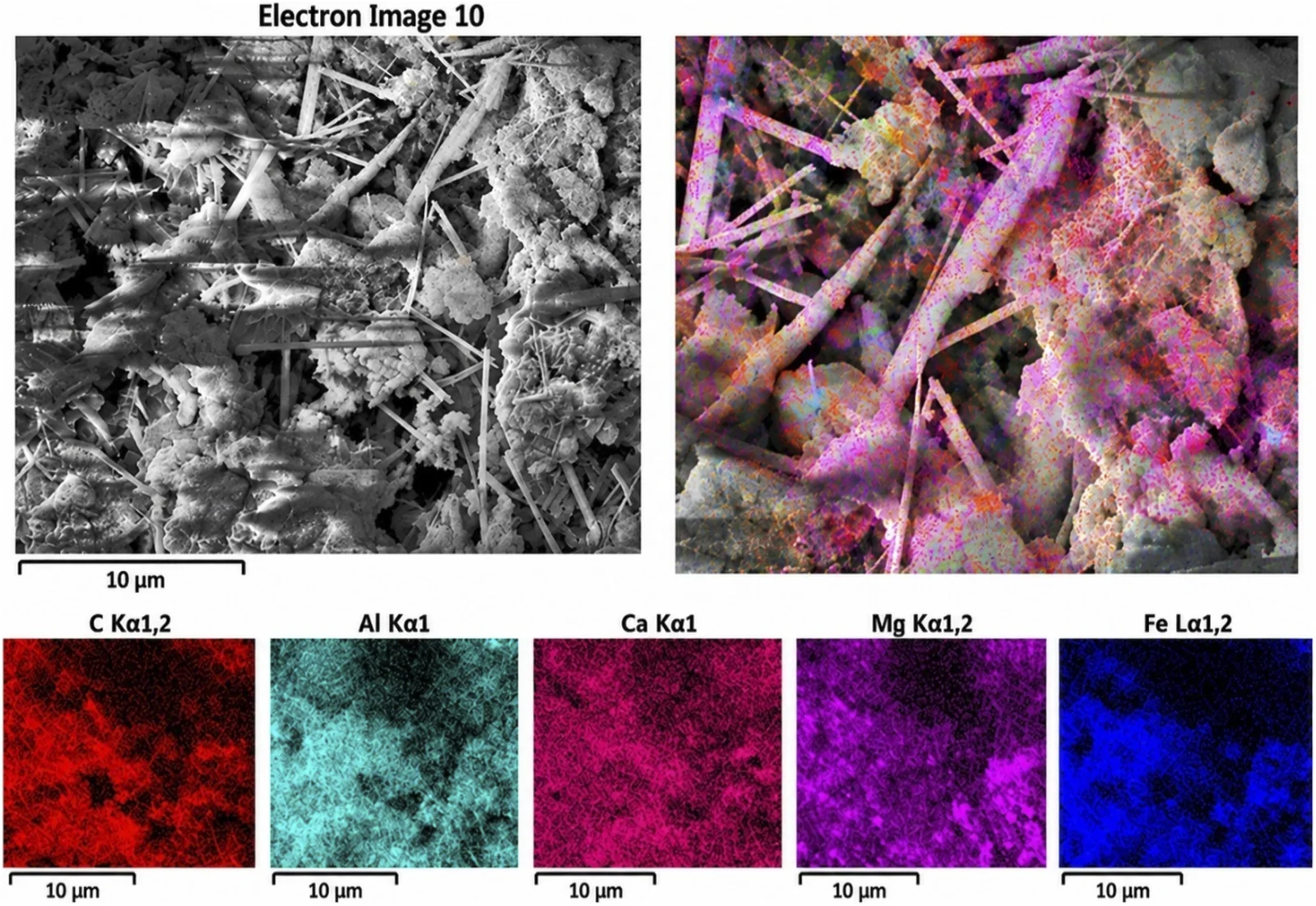
EDS elemental mapping of bottom ash cured with the Fe_2_O_3_-modified zeolite/Ca_3_(PO_4_)_2_ composite containing 8 wt% Fe_2_O_3_ (M3).

The untreated bottom ash (M0) consists of a heterogeneous assemblage of irregular angular particles interspersed with smoother spherical grains (Fig. 10a). Localized agglomeration and relatively compact surface regions are readily observed, reflecting the intrinsic heterogeneity of municipal solid waste incineration bottom ash. After curing with natural zeolite (M1), lamellar particles characteristic of clinoptilolite become readily recognizable within the matrix, accompanied by a less compact particle arrangement and more pronounced interparticle spaces (Fig. 10b). These changes suggest that incorporation of the zeolite support modifies the external morphology of the cured material while maintaining a relatively open particle assembly.

Introducing the α-Fe_2_O_3_-modified zeolite leads to further changes in surface morphology. At 4 wt% Fe_2_O_3_ (M2), the particle arrangement remains relatively heterogeneous, and only limited development of interconnected voids is evident (Fig. 10c). Increasing the Fe_2_O_3_ loading to 8 wt% (M3) produces a noticeably rougher surface together with a more open and interconnected microstructure (Fig. 10d). Localized surface deposits appear to be present, although substantial portions of the original particles remain exposed. This intermediate morphology is consistent with a balance between deposition of the iron-containing phase and preservation of an open particle assembly, mirroring the maximum surface area and pore volume observed by BET. Further increasing the Fe_2_O_3_ loading to 12 wt% (M4) yields a denser particle assembly in which compact regions become more evident (Fig. 10e). One plausible explanation is progressive filling of interparticle voids as the amount of deposited iron-containing material increases. Such densification may also reduce the accessibility of internal surface regions, providing a plausible explanation for the lower specific surface area measured by BET at the highest Fe_2_O_3_ loading.

The compositional changes revealed by EDS follow the same general trend (Fig. 11).

Oxygen, silicon, calcium, carbon and aluminum remain the dominant elements throughout the curing system, reflecting contributions from both the bottom ash and the zeolitic support. Relative to the untreated material, incorporation of the α-Fe_2_O_3_-coated composite results in an increased Fe signal, while calcium and phosphorus are also detected following addition of Ca_3_(PO_4_)_2_. These compositional changes are compatible with successful retention of the added materials within the cured matrix. Because EDS provides elemental rather than phase-specific information, the coexistence of Fe, Ca and P should not be interpreted as direct evidence for particular mineral phases or the reactions through which they formed.

Elemental mapping further clarifies the spatial distribution of the principal elements within the cured matrix (Fig. 12).

Iron is distributed across most of the analyzed region without obvious micron-scale enrichment, whereas calcium, silicon, aluminum and phosphorus likewise exhibit broad spatial distributions throughout the matrix. Although nanoscale dispersion cannot be resolved by this technique, the absence of distinct Fe-rich regions suggests that the iron-containing phase remains broadly dispersed rather than concentrated into isolated micron-sized domains. This distribution is consistent with the XRD and FTIR results, which collectively suggest that curing promotes structural evolution without disrupting the underlying aluminosilicate framework. The SEM, EDS, and elemental mapping results reveal progressive microstructural and compositional evolution of the curing matrix following incorporation of the α-Fe_2_O_3_-modified zeolite composites. These observations provide the structural context for interpreting the changes in PTE fractionation discussed in the following section.

### 3.3. Time-dependent structural evolution during curing

#### 3.3.1. Time-dependent XRD evolution

Based on the characterization results presented in Sections 3.1 and 3.2, the zeolite/α-Fe_2_O_3_ composite containing 8 wt% Fe_2_O_3_ was selected for the curing study because it exhibited the most favorable structural and textural properties. Structural evolution of the cured bottom ash was subsequently monitored over four weeks (W1–W4) using XRD and FTIR spectroscopy (Fig. 13 and 14).

**Fig. 13:**
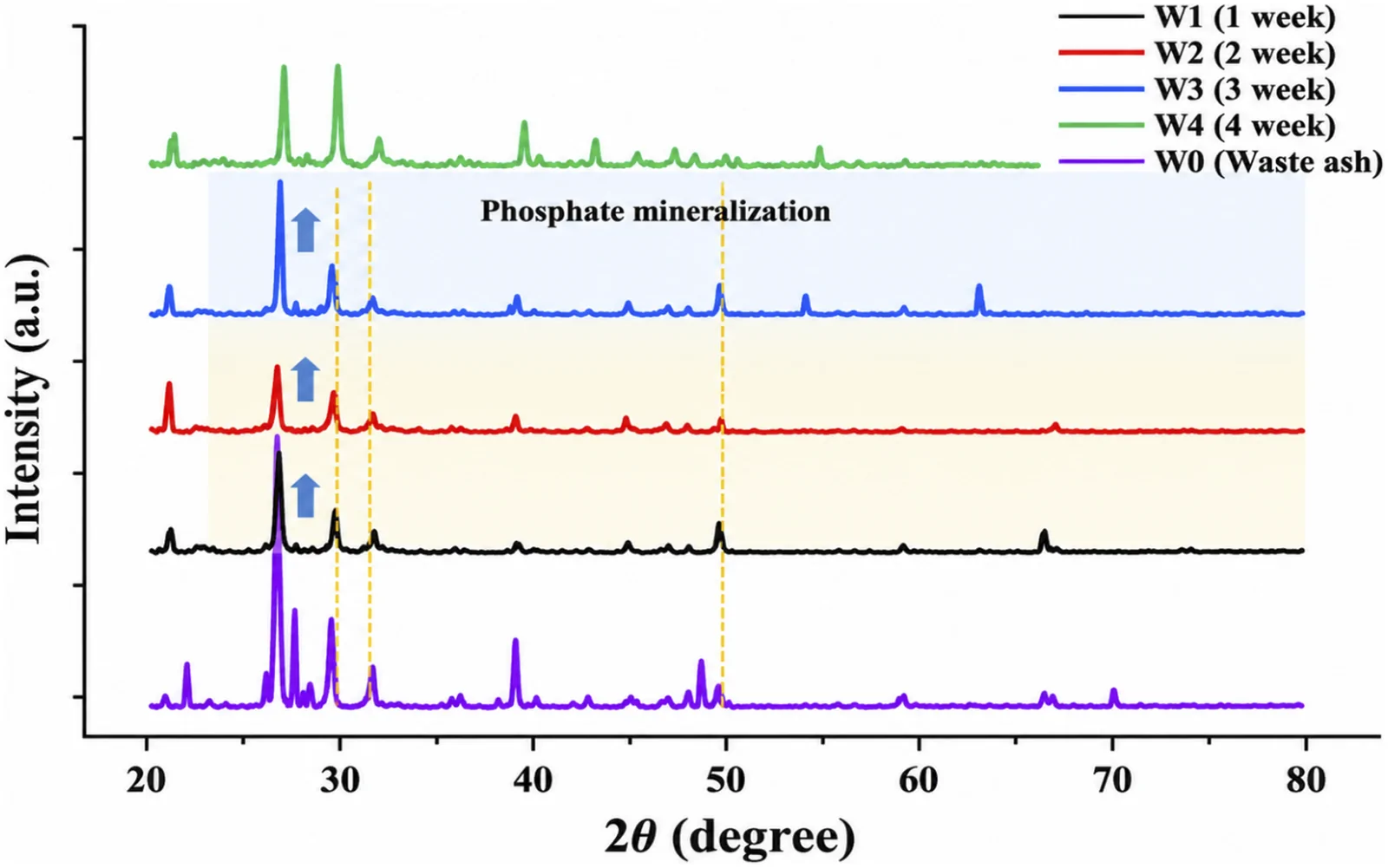
XRD patterns of untreated bottom ash (M0) and bottom ash cured with the Fe_2_O_3_-modified zeolite composite containing 8 wt% Fe_2_O_3_ after 1, 2, 3, and 4 weeks of curing.

**Fig. 14:**
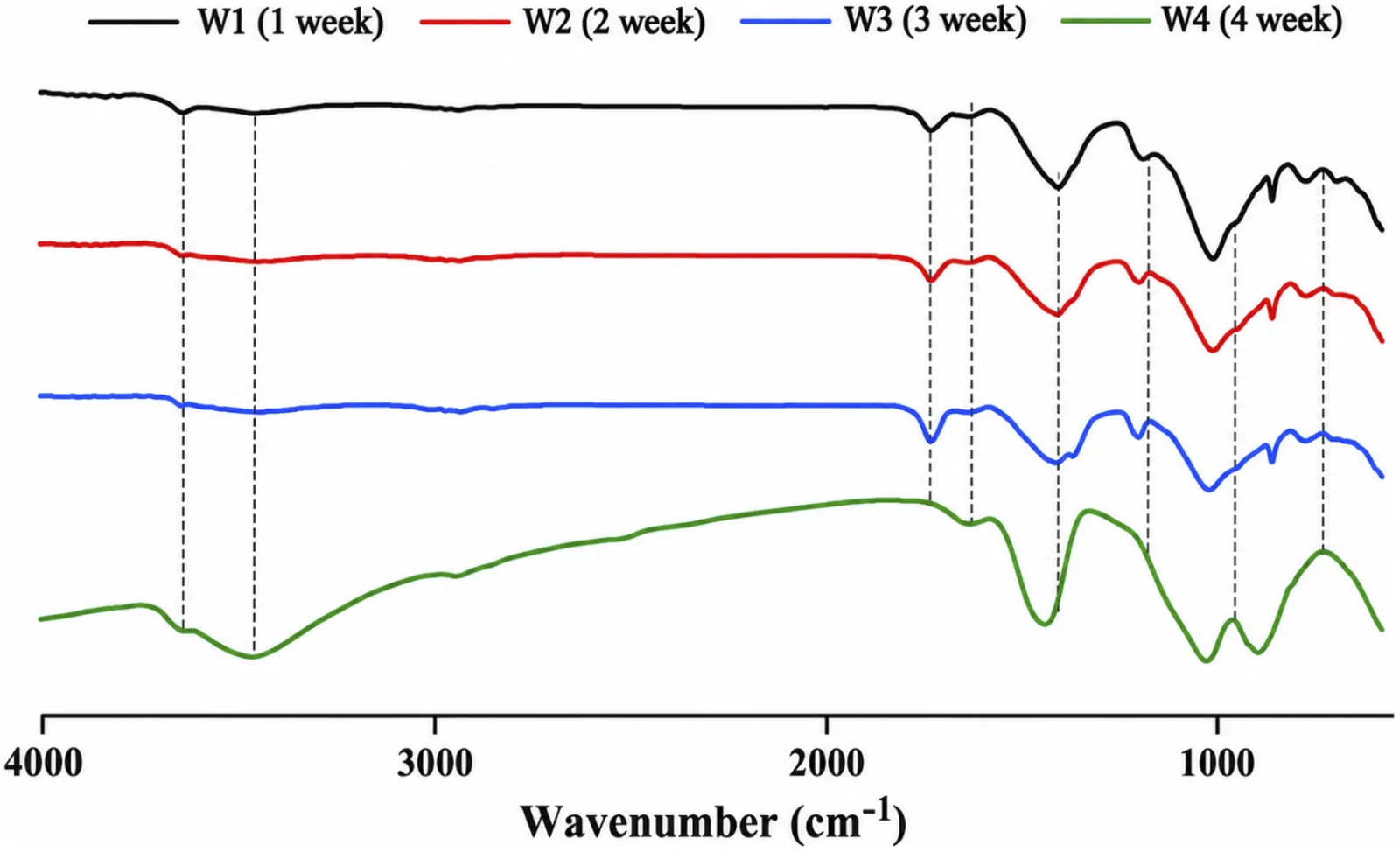
FTIR spectra of municipal solid waste incineration (MSWI) bottom ash treated with the zeolite/α-Fe_2_O_3_ composite (8 wt% Fe_2_O_3_) after 1, 2, 3, and 4 weeks of curing. Dashed lines highlight the principal vibrational regions that evolved during the curing process.

Only modest changes are observed after the first week (W1), with most reflections remaining comparable to those of the untreated ash (W0), suggesting that crystallographic rearrangement remains in its initial stage. By W2, several additional diffraction features become discernible, particularly within the 29–33° (2*θ*) region, accompanied by subtle variations in peak intensity.^40^ Their emergence coincides with the stage at which curing has progressed sufficiently for measurable crystallographic reorganization to occur. Because several mineral phases may contribute to overlapping reflections within this angular range, these features are more appropriately interpreted as evidence of mineral assemblage evolution than as direct identification of newly formed phases.

Further changes occur during W3 and W4, where several reflections become better resolved and peak sharpening is evident around 29–30° and 51–52° (2*θ*).^41^ Such changes may reflect increasing crystallinity, changes in relative phase abundance, or continued crystal growth during curing rather than exclusively indicating formation of new crystalline phases. The comparatively minor differences between W3 and W4 further suggest that the rate of crystallographic reorganization decreases as the curing system approaches a relatively stable structural state.

#### 3.3.2. Time-dependent FTIR evolution

FTIR spectroscopy provides complementary insight into changes occurring within the local chemical environment during curing (Fig. 14).

A broad hydroxyl stretching band at 3650–3400 cm^−1^ gradually intensifies from W1 to W4. Compared with the limited spectral variation observed during the first week, substantially greater changes appear after W2, mirroring the period during which XRD first records appreciable crystallographic modification. The observed spectral evolution is consistent with progressive hydration of reactive mineral surfaces, although FTIR provides only indirect evidence because it probes local bonding environments rather than crystal structures. Because FTIR probes short-range bonding environments rather than crystal structures, these spectral changes should be regarded as indirect evidence of evolving surface chemistry. The band at 1750–1630 cm^−1^, attributed to molecularly bound water and H–O–H bending vibrations, also becomes increasingly pronounced with curing time,^41^ indicating continuing adjustment of the hydration environment surrounding the solid matrix. Within the aluminosilicate region (1180–1220 cm^−1^), gradual band broadening and slight distortion become evident from W2 onward.^41^ These spectral variations are more plausibly associated with modification of the surrounding Si–O–Si/Si–O–Al bonding environment than with reconstruction of the zeolitic framework, consistent with the XRD observations that the principal structural characteristics remain preserved throughout curing.

Additional evolution within the 1400–1500, 950–1050, and 550–600 cm^−1^ regions^42,43^ is compatible with increasing contributions from carbonate-containing environments, phosphate-related vibrations, and Fe–O lattice vibrations, respectively. The gradual strengthening of carbonate-related bands also agrees with continued carbonation under ambient curing conditions. Owing to substantial overlap among these absorptions, the spectral features are more appropriately interpreted in combination rather than assigned individually to specific reaction products.

#### 3.3.3. Structural evolution during curing

Considered together, the XRD and FTIR results describe a coherent sequence of structural development throughout the four-week curing period. Only modest crystallographic and spectroscopic changes occur during the first week, whereas the interval between W2 and W3 corresponds to the most pronounced structural reorganization, indicating that curing becomes substantially more advanced during this stage. The comparatively small differences observed between W3 and W4 suggest that the system gradually approaches structural equilibrium as curing proceeds.

The two techniques provide complementary perspectives on the same process: XRD follows changes in long-range crystallographic order, whereas FTIR captures adjustments within local bonding and hydration environments. Together, these results provide a consistent description of the evolving solid matrix and establish the physicochemical framework for interpreting the sequential extraction results presented in the following section. These complementary datasets collectively provide mechanistic insight into the stabilization of PTEs.

### 3.4. Evaluation of PTE stabilization by sequential extraction

BET, XRD, FTIR and SEM collectively characterized the structural evolution of the Fe_2_O_3_-modified zeolite composites during curing. These techniques, however, cannot distinguish whether potentially toxic elements (PTEs) remain in mobile forms or are transformed into geochemically stable fractions. To address this limitation, Tessier sequential extraction coupled with ICP-OES was employed to quantify metal partitioning among the operationally defined fractions (F1–F5). This approach enables direct assessment of changes in metal speciation, providing the basis for the mechanistic interpretation of PTE stabilization presented below.

#### 3.4.1. Effect of Fe_2_O_3_ loading on the speciation and stabilization of PTEs in zeolite-based composites

Sequential extraction provides a more environmentally relevant assessment of the immobilization of PTEs than bulk elemental analysis because environmental risk is governed more strongly by chemical speciation than by total elemental concentration. Combined with ICP-OES, it enables direct evaluation of how Fe_2_O_3_ loading modifies the distribution of metals among operationally defined fractions (F1–F5). The resulting fraction distributions are summarized in Fig. 15 and Table S4 (SI), while their overall relationships are further examined using hierarchical cluster analysis (Fig. 16).

**Fig. 15:**
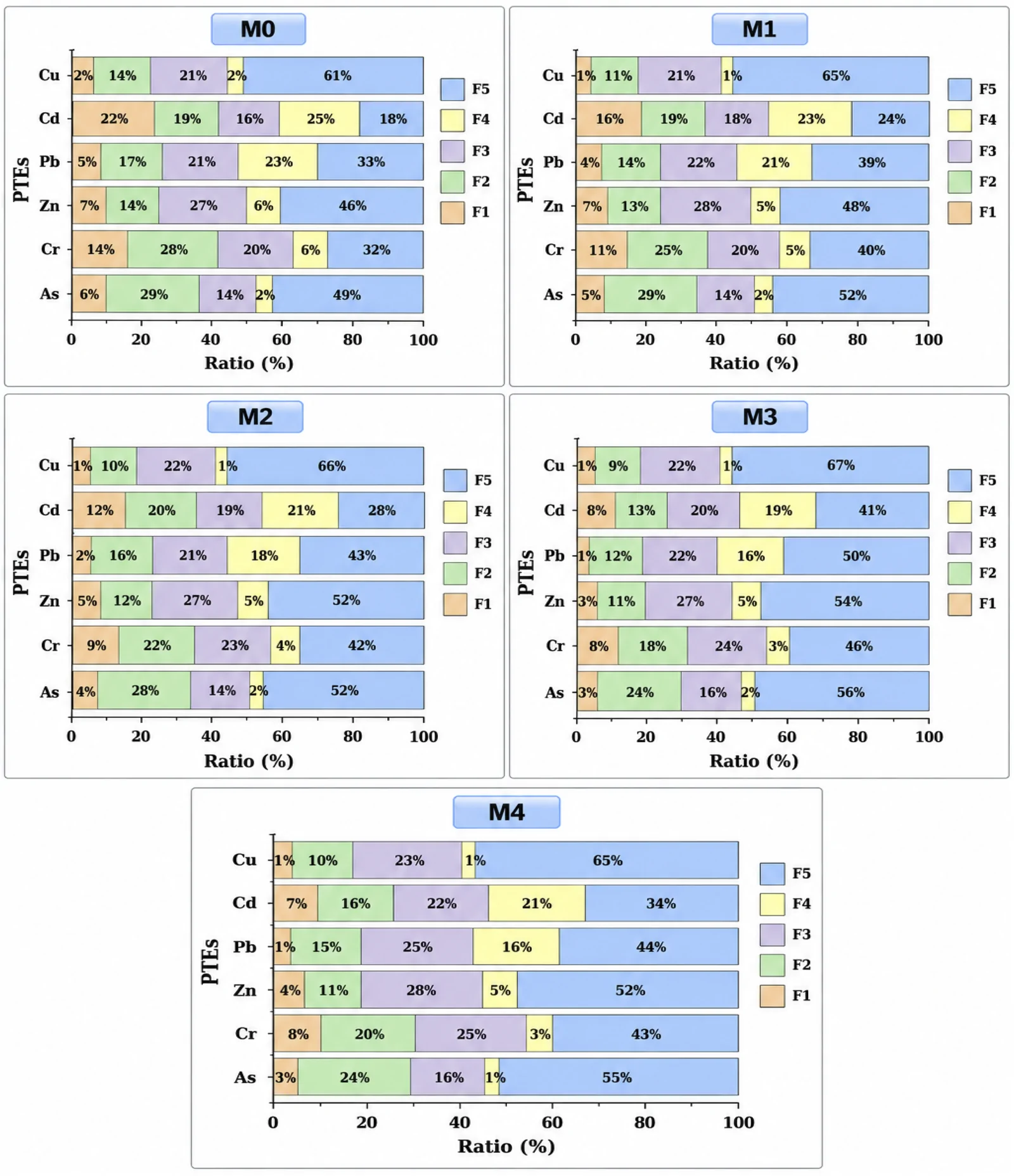
Distribution of chemical fractions (%) of potentially toxic elements (PTEs) in municipal solid waste incineration bottom ash after curing. M0 represents untreated bottom ash, M1 corresponds to bottom ash amended with natural zeolite, and M2–M4 correspond to bottom ash amended with zeolite/Fe_2_O_3_ composites containing 4, 8 and 12 wt% Fe_2_O_3_, respectively.

**Fig. 16:**
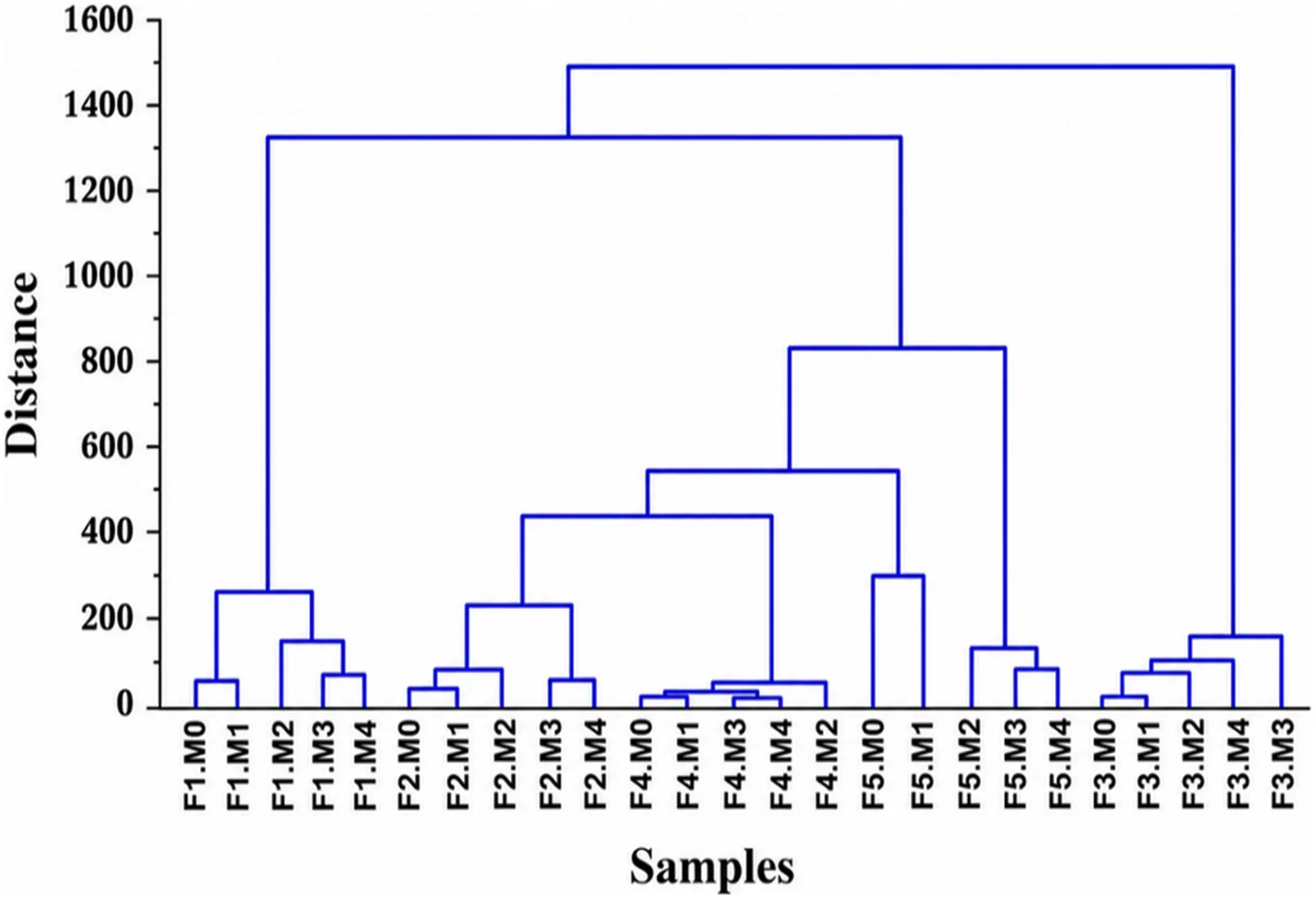
Hierarchical clustering of chemical fractions (F1–F5) of potentially toxic elements (PTEs) in municipal solid waste incineration bottom ash after curing. M0 represents untreated bottom ash; M1 denotes bottom ash amended with natural zeolite and Ca_3_(PO_4_)_2_; M2–M4 denote bottom ash amended with Fe_2_O_3_-modified zeolite containing 4, 8, and 12 wt% Fe_2_O_3_, respectively, with Ca_3_(PO_4_)_2_ included in all amended treatments.

##### 3.4.1.1. Untreated bottom ash

Untreated bottom ash (M0) contained substantial exchangeable and carbonate-bound fractions (F1 + F2), indicating high remobilization potential (Fig. 15). The combined mobile fraction reached 42% for Cr, 41% for Cd and 35% for As, whereas Pb (22%), Zn (21%) and Cu (16%) exhibited considerably lower mobility. This distribution is consistent with the known geochemical behaviour of these metals. Cd^2+^ generally forms weak outer-sphere associations and is therefore readily displaced by competing cations. In contrast, As and Cr mobility is largely governed by redox and solution chemistry, whereas Pb and Cu preferentially undergo inner-sphere complexation or precipitation.

##### 3.4.1.2. Natural zeolite

Natural zeolite (M1) induced only limited changes in metal speciation. A modest decrease in F1–F2 accompanied by a slight increase in F5 indicates that ion exchange reduced short-term mobility without substantially altering long-term geochemical partitioning. This behaviour reflects the predominance of electrostatic exchange within the aluminosilicate framework, which provides fewer highly reactive coordination sites than Fe_2_O_3_-modified zeolite.

##### 3.4.1.3. Fe_2_O_3_-mediated stabilization mechanism

Introducing Fe_2_O_3_ substantially changed the immobilization pathway. Increasing the Fe_2_O_3_ loading from 4% (M2) to 8% (M3) markedly decreased F1–F2 while increasing F5 for nearly all investigated metals (Fig. 15). This systematic shift is consistent with a contribution from changes in interfacial chemistry, rather than being attributable to physical adsorption alone. Reactive Fe-containing surface sites may have contributed to surface complexation and ligand-exchange processes, whereas Fe_2_O_3_–zeolite interfaces may also have favoured the formation of less soluble hydroxide-, phosphate-, or Fe-associated phases during curing.

Cadmium exhibited the strongest response, with the combined F1–F2 fraction decreasing from 41% in M0 to 32% in M2 and 21% in M3. This trend is consistent with contributions from Fe-associated surface interactions and/or precipitation-related retention, although the present data do not distinguish these pathways directly. Chromium displayed a comparable but weaker response, plausibly reflecting stabilization through hydroxide formation, Fe-rich phases and ligand exchange involving chromate species.

##### 3.4.1.4. Metal-specific behaviour and optimum Fe_2_O_3_ loading

Pb and As followed distinct yet complementary immobilization patterns. The decline in mobile Pb fractions is consistent with stronger interactions with Fe-containing surfaces together with phosphate-associated processes, whereas As redistribution is consistent with interactions involving surface Fe sites. In contrast, Cu and Zn exhibited more limited redistribution among the operationally defined fractions than the other investigated PTEs. For Cu, this behaviour is consistent with its initially stable partitioning in MSWI bottom ash. Our previous study showed that approximately 68–70% of Cu was associated with the residual fraction, indicating that a substantial proportion of Cu was already present in relatively stable solid-phase associations before treatment.^2^ XAS, XRD, and geochemical modelling studies have likewise reported multiple mineral-associated forms of Cu in MSWI bottom ash.^44^ Cu-dissolved organic carbon interactions have also been reported in bottom-ash leachates, suggesting that organic associations may additionally influence Cu mobility.^45^ These pre-existing associations may have limited the fraction of Cu available for further redistribution during curing. The limited Cu response should therefore not be interpreted as evidence of weak affinity for Fe-containing surfaces, as Cu has been reported to form inner-sphere complexes with iron (oxyhydroxide/oxide) surfaces.^46^ The limited Cu redistribution is therefore interpreted primarily in the context of its initially stable partitioning and the consequently limited pool available for further redistribution under the investigated conditions.

Within the investigated loading range, 8 wt% α-Fe_2_O_3_ provided the best overall stabilization performance. Increasing the loading from 4 to 8 wt% enhanced the redistribution of PTEs from the labile F1–F2 fractions towards less labile fractions, whereas further increasing the loading to 12 wt% provided no additional stabilization benefit. The 8 wt% Fe_2_O_3_-modified zeolite also exhibited the most favourable specific surface area and pore volume, whereas the 12 wt% composite showed lower textural parameters together with more pronounced surface deposition and particle aggregation. These converging observations support a loading-dependent association among Fe_2_O_3_ loading, textural accessibility, and PTE fractionation within the investigated range. However, they do not establish a quantitative or causal relationship between individual textural parameters and PTE redistribution. Given the limited number of Fe_2_O_3_ loading levels investigated, 8 wt% is therefore considered the best-performing loading within the tested range rather than an absolute optimum. Hierarchical cluster analysis was subsequently used to further examine the loading-dependent fractionation patterns (Fig. 16).

##### 3.4.1.5. Hierarchical cluster analysis and mechanistic implications

Hierarchical cluster analysis independently supported the mechanism inferred from sequential extraction (Fig. 16). Exchangeable and carbonate-bound fractions (F1–F2) clustered as the most labile domain, whereas organic-bound and residual fractions (F4–F5) formed the most stable geochemical sink. The Fe–Mn oxide fraction (F3) occupied an intermediate position, consistent with its role as a transitional host during fraction redistribution. This clustering is fully consistent with the fraction redistribution revealed by sequential extraction.

Among the investigated materials, M3 exhibited the greatest separation from untreated ash, whereas M2 occupied an intermediate position and M4 shifted slightly toward the untreated material. This clustering pattern provides independent multivariate support for the superior fractionation response of M3 relative to the other amended samples. Sequential extraction cannot resolve molecular-scale coordination directly. Nevertheless, when interpreted together with the structural characterization results, it consistently demonstrates that Fe_2_O_3_-modified zeolite promotes redistribution of metals from labile to persistent geochemical fractions.

#### 3.4.2. Time-dependent evolution of potentially-toxic-element speciation during curing with zeolite/Fe_2_O_3_/Ca_3_(PO_4_)_2_

Curing time markedly influenced the chemical speciation of PTEs in the zeolite/Fe_2_O_3_ (8 wt%)/Ca_3_(PO_4_)_2_ system, leading their progressive redistribution from mobile to more geochemically stable fractions (Table 3 and Fig. 17).

**Table 3:** Concentrations of As, Cr, Zn, Pb, Cd, and Cu in sequential extraction fractions (F1–F5) of municipal solid waste incineration bottom ash during curing with the zeolite/Fe_2_O_3_/Ca_3_(PO_4_)_2_ composite. Values are expressed as mean (*n* = 3), SD, and RSD (%)

Chemical fraction(s)	Concentration (mg kg^−1^)						pH	EC
	As	Cr	Zn	Pb	Cd	Cu		
**Untreated ash (W0)**								
F1	6.192	7.225	160.1	3.292	23.73	17.36	5.6 ± 0.12	2.5 ± 0.04
SD	0.130	0.315	0.039	0.788	0.062	3.922		
RSD%	2.096	4.361	1.897	3.113	3.304	4.998		
F2	5.217	2.042	441.8	58.12	62.43	71.25		
SD	0.245	0.039	16.79	1.162	1.061	1.567		
RSD%	4.693	1.897	3.800	1.999	1.700	2.199		
F3	9.450	25.31	245.8	46.78	60.15	88.76		
SD	0.468	0.788	6.340	1.768	3.029	3.234		
RSD%	4.956	3.113	2.579	3.699	5.036	3.643		
F4	2.065	1.890	89.18	28.80	64.40	44.91		
SD	0.079	0.062	4.103	0.403	1.609	0.808		
RSD%	3.828	3.304	4.600	1.401	2.500	1.800		
F5	12.30	78.46	444.9	81.88	107.9	1131		
SD	0.487	3.922	8.454	3.931	3.670	7.937		
RSD%	3.959	4.998	1.900	4.801	3.400	0.702		

**Week 1 (W1)**								
F1	5.604	6.107	146.4	2.799	19.575	14.08	6.5 ± 0.23	2.8 ± 0.05
SD	0.239	0.308	5.522	0.137	0.925	0.558		
RSD%	4.272	5.048	3.772	4.898	4.724	3.960		
F2	4.841	1.745	368.3	48.59	55.192	65.98		
SD	0.232	0.053	8.989	2.252	2.785	3.167		
RSD%	4.787	3.061	2.441	4.636	5.046	4.801		
F3	10.02	26.88	255.5	52.34	61.434	95.10		
SD	0.270	1.139	6.753	2.207	3.085	4.599		
RSD%	2.695	4.238	2.643	4.217	5.022	4.836		
F4	2.017	1.945	92.32	29.48	67.697	48.58		
SD	0.017	0.077	4.500	1.498	2.825	2.332		
RSD%	0.860	3.955	4.874	5.080	4.173	4.801		
F5	13.19	83.76	488.0	103.4	123.3	1173		
SD	0.131	4.161	5.103	4.942	5.367	10.603		
RSD%	0.994	4.968	1.046	4.779	4.353	0.904		

**Week 2 (W2)**								
F1	3.939	4.828	140.2	1.936	14.33	12.19	7.1 ± 0.11	3.2 ± 0.02
SD	0.142	0.218	6.182	0.070	0.695	0.359		
RSD%	3.601	4.519	4.408	3.599	4.849	2.948		
F2	4.023	1.131	349.9	38.24	46.27	57.50		
SD	0.105	0.053	8.996	1.931	1.585	2.280		
RSD%	2.619	4.682	2.570	5.049	3.425	3.964		
F3	10.82	28.82	260.1	54.38	64.47	93.37		
SD	0.459	1.319	6.578	2.396	2.487	2.840		
RSD%	4.241	4.577	2.529	4.407	3.858	3.042		
F4	2.200	2.003	93.66	30.91	69.69	50.84		
SD	0.093	0.097	4.504	0.894	2.732	2.205		
RSD%	4.207	4.847	4.808	2.892	3.921	4.337		
F5	16.27	85.35	513.8	113.1	137.3	1191		
SD	0.792	4.064	6.185	3.671	6.149	6.799		
RSD%	4.870	4.761	1.204	3.246	4.478	0.571		

**Week 3 (W3)**								
F1	3.233	2.819	128.7	1.261	9.392	9.328	7,5 ± 0.24	3.3 ± 0.01
SD	0.097	0.111	5.992	0.034	0.458	0.421		
RSD%	3.000	3.922	4.656	2.717	4.877	4.518		
F2	3.194	1.035	323.3	22.79	29.08	47.11		
SD	0.096	0.030	8.602	0.925	1.092	1.872		
RSD%	3.006	2.902	2.661	4.061	3.753	3.974		
F3	8.970	26.85	256.8	52.16	63.14	90.57		
SD	0.269	1.309	8.487	2.541	2.439	4.308		
RSD%	2.999	4.875	3.305	4.871	3.863	4.757		
F4	2.174	2.015	97.98	31.99	72.38	51.97		
SD	0.065	0.096	4.311	1.480	2.947	2.351		
RSD%	2.990	4.766	4.400	4.626	4.071	4.525		
F5	20.87	91.51	543.1	122.3	158.0	1212		
SD	0.630	4.131	16.30	5.590	5.397	15.18		
RSD%	3.019	4.514	3.001	4.572	3.408	1.253		

**Week 4 (W4)**								
F1	3.115	2.785	126.4	1.223	9.287	9.303	7.7 ± 0.45	
SD	0.084	0.117	3.800	0.037	0.257	0.249		
RSD%	2.706	4.187	3.006	3.025	2.767	2.680		
F2	3.149	1.015	319.4	20.66	27.94	48.02		
SD	0.142	0.034	9.600	0.648	1.181	2.342		
RSD%	4.507	3.354	3.006	3.138	4.229	4.879		
F3	8.893	25.46	253.7	50.58	62.46	88.96		
SD	0.432	1.154	6.568	2.483	1.870	3.543		3.5 ± 0.03
RSD%	4.854	4.532	2.589	4.910	2.994	3.983		
F4	2.110	1.994	96.79	30.73	71.24	52.52		
SD	0.095	0.097	3.834	0.739	2.806	2.313		
RSD%	4.486	4.853	3.961	2.404	3.939	4.405		
F5	21.56	92.06	547.2	124.6	162.5	1214		
SD	0.650	3.599	9.940	5.396	4.900	7.937		
RSD%	3.015	3.910	2.997	4.330	3.015	0.654		

**Fig. 17:**
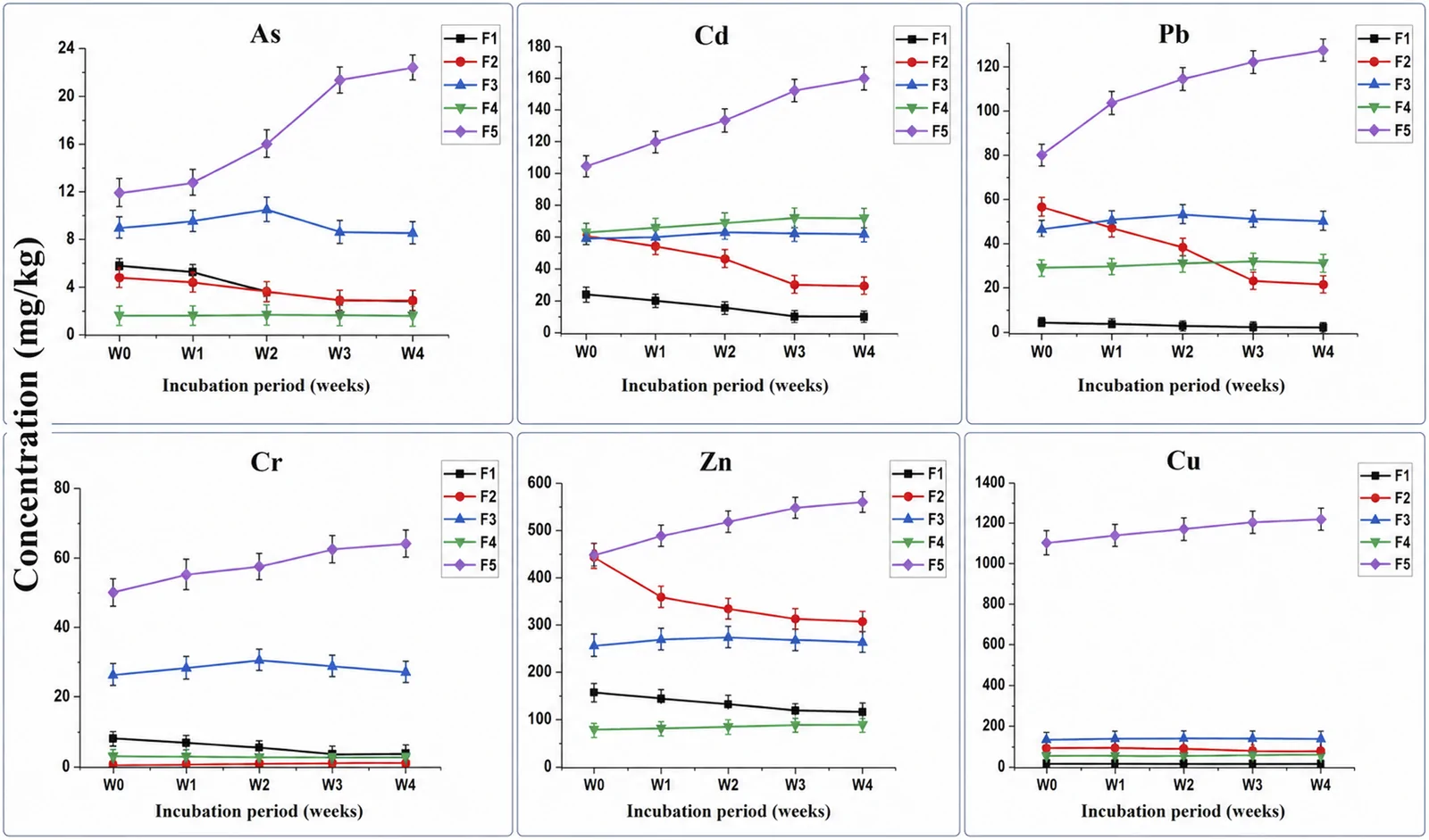
Evolution of the chemical fractions (%) of PTEs in municipal solid waste incineration bottom ash during curing with the zeolite/Fe_2_O_3_ (8 wt%)/Ca_3_(PO_4_)_2_ composite. W0 represents untreated bottom ash, whereas W1–W4 correspond to bottom ash cured for 1–4 weeks, respectively.

##### 3.4.2.1. Evolution of pH and electrical conductivity during curing

The pH of the bottom ash increased progressively during curing, from 5.6 at W0 to 7.7 after four weeks (W4), indicating a gradual shift in the system from mildly acidic towards mildly alkaline conditions following amendment with zeolite, α-Fe_2_O_3_, and Ca_3_(PO_4_)_2_. The observed pH changes are relevant to PTE stabilization because pH can influence metal speciation, mineral-surface charge, and the relative contribution of sorption, surface complexation, and precipitation processes. For predominantly cationic PTEs such as Cd, Pb, Zn, and Cu, the shift towards higher pH may favor their retention through enhanced interactions with mineral surfaces and/or the formation of less soluble solid phases. Accordingly, the progressive increase in pH may have contributed to the redistribution of PTEs from the more labile F1–F2 fractions towards the more stable fractions during curing. This interpretation is consistent with the observed fractionation trends, although the present data do not allow the individual contributions of surface sorption, complexation, and precipitation to be distinguished.

The electrical conductivity (EC) increased concurrently from 2.5 ± 0.04 mS cm^−1^ at W0 to 3.5 ± 0.03 mS cm^−1^ at W4, corresponding to an increase of approximately 40%. The increase in EC reflects changes in the dissolved electrolyte environment during curing and may be associated with dissolution, ion exchange, and progressive re-equilibration between the solid and aqueous phases following amendment. EC, however, reflects the overall conductivity of the dissolved electrolyte phase and is not a PTE-specific indicator of dissolution or mobility. Notably, the increase in EC occurred alongside a general decline in the labile F1 and F2 fractions and an increase in the residual F5 fraction. Thus, the increase in EC was not accompanied by greater partitioning of PTEs into the operationally defined labile fractions. Taken together, the concurrent evolution of pH and EC reflects changes in the physicochemical environment during curing, while the decline in F1–F2 alongside the increase in F5 is consistent with a progressive redistribution of PTEs towards less labile fractions within the bottom-ash–amendment system.

Across all investigated metals, the exchangeable (F1) and carbonate-bound (F2) fractions progressively declined, whereas the residual fraction (F5) increased throughout the four-week curing period, indicating continuous transformation from labile to geochemically stable forms. This temporal redistribution indicates that immobilization progressively evolved during curing rather than occurring instantaneously through surface adsorption alone, consistent with the structural evolution revealed by XRD and FTIR (Section 3.3). Based on the reduction in the combined mobile fraction (F1 + F2), stabilization followed the sequence Pb > Cd > As > Cr > Zn > Cu, highlighting the strong influence of intrinsic metal geochemistry on redistribution behaviour. Pb and Cd responded most rapidly, whereas Zn and Cu exhibited comparatively slower changes, indicating that stabilization pathways differed among individual metals.

Arsenic and chromium underwent continuous redistribution from mobile to residual fractions throughout curing. For As, F1 and F2 decreased by approximately 50% and 40%, respectively, whereas F5 increased from 12.30 to 21.56 mg kg^−1^ after four weeks. Chromium exhibited a comparable trend, with reductions of 61% in F1 and 50% in F2 accompanied by gradual enrichment of F5. This behaviour is consistent with progressive stabilization on Fe-rich surfaces. Plausible pathways include ligand exchange, specific adsorption and subsequent incorporation into Fe-containing secondary phases during curing. Although these processes were not directly resolved, they provide a chemically plausible explanation for the sustained shift toward less mobile fractions.

Pb and Cd exhibited the greatest redistribution. After four weeks, exchangeable Pb and Cd decreased by approximately 63% and 61%, respectively, whereas their carbonate-bound fractions declined by more than 55%, accompanied by substantial enrichment of F5. This response is consistent with the strong tendency of Pb^2+^ and Cd^2+^ to form sparingly soluble phosphate phases while simultaneously undergoing stable complexation on Fe oxide surfaces. The combined contribution of these processes plausibly explains their faster stabilization relative to the remaining metals.

Zn responded more gradually despite its relatively high initial mobility. The smaller reductions in F1 (21%) and F2 (28%) suggest slower transformation into stable solid phases, possibly reflecting the comparatively higher solubility of Zn-bearing products under the present curing conditions. By contrast, Cu exhibited only minor redistribution because it was already predominantly associated with the residual fraction before treatment, leaving limited scope for further stabilization. These contrasting responses emphasize that immobilization depends not only on composite reactivity but also on the inherent geochemical characteristics of individual metals. The most pronounced redistribution occurred between Weeks 2 and 3 for nearly all investigated metals, whereas only marginal changes were observed thereafter. This interval coincided with the most evident structural evolution of the curing matrix identified by XRD and FTIR (Section 3.3), suggesting that mineral reorganization and metal redistribution progressed concurrently. During the early curing stage, abundant reactive surface sites favoured adsorption, complexation and precipitation. As curing proceeded, the observed changes in PTE fractionation became progressively smaller, suggesting that the system approached a comparatively stable partitioning state by Week 4. This observation is restricted to the investigated four-week curing period and does not establish that further curing would produce negligible additional changes.

##### 3.4.2.2. Principal component analysis of time-dependent speciation evolution

To complement the sequential extraction results, principal component analysis (PCA) was applied to the time-resolved speciation dataset (Fig. 18). The first two principal components explained approximately 94% of the total variance (PC1: 76.7–76.9%; PC2: 17.6–17.8%). The high explained variance indicates a well-defined trajectory of chemical-speciation evolution during curing and provides a multivariate framework for interpreting the redistribution of PTEs among the operationally defined fractions (F1–F5). The PCA loading plot separated the operational fractions according to their environmental mobility. Exchangeable and carbonate-bound fractions (F1–F2), together with the transitional Fe–Mn oxide fraction (F3), clustered along PC1, whereas the residual fraction (F5) occupied a distinct position toward PC2, reflecting its role as the least-extractable fraction. The score trajectories showed progressive shifts of As, Cr, Pb and Cd away from the mobile-fraction domain toward the F5 domain as curing proceeded, consistent with the redistribution identified by sequential extraction.

**Fig. 18:**
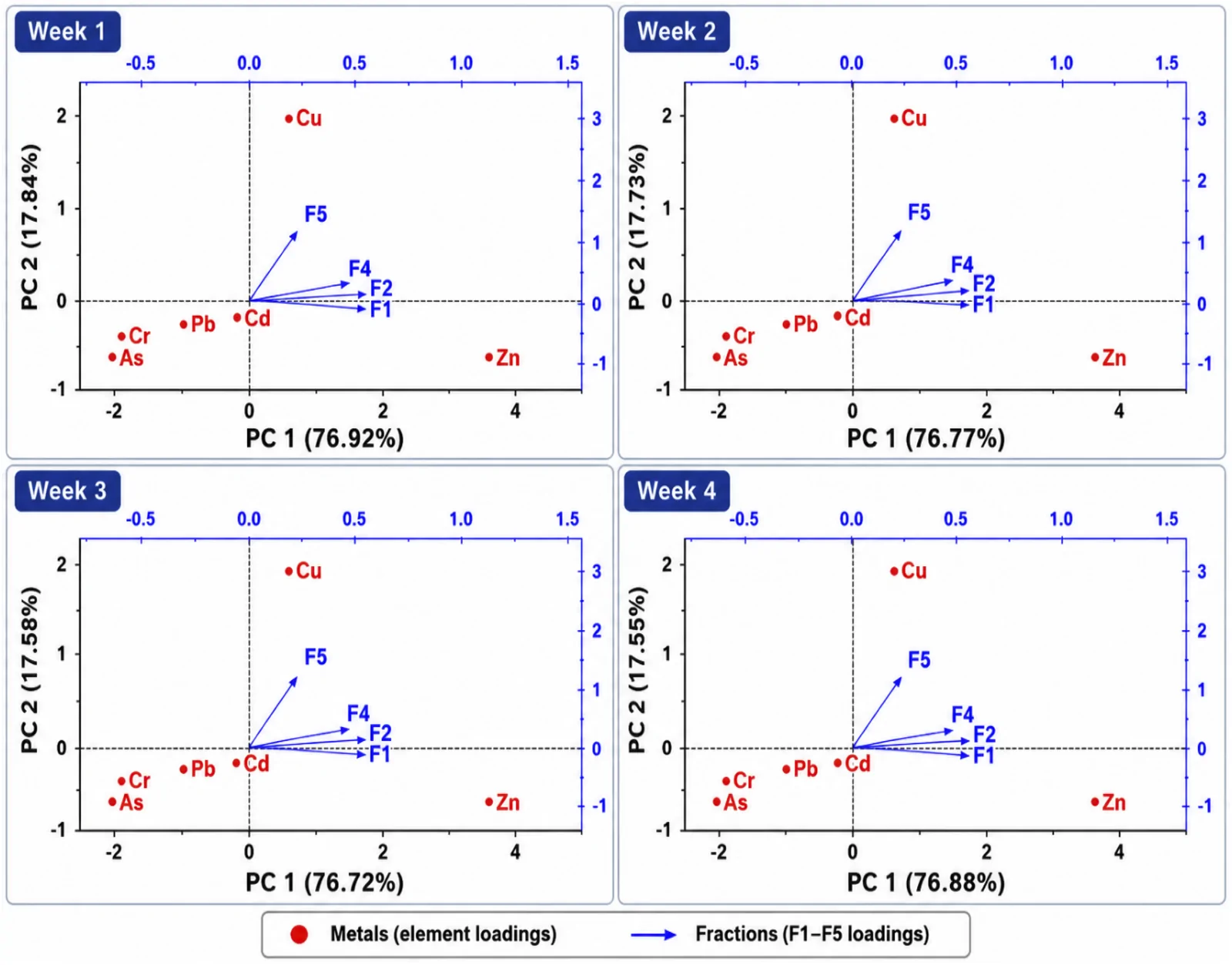
Principal component analysis (PCA) of the chemical speciation of potentially toxic elements (PTEs) in municipal solid waste incineration bottom ash during curing with the zeolite/Fe_2_O_3_ (8 wt%)/Ca_3_(PO_4_)_2_ composite. PCA was constructed using the operationally defined fractions (F1–F5).

In contrast, Zn exhibited a shorter trajectory, consistent with its comparatively slower redistribution during curing, whereas Cu remained comparatively stable because it was already predominantly associated with the residual fraction before treatment. The close agreement between PCA and sequential extraction provides independent multivariate support for the proposed stabilization mechanism. Together, these complementary analyses consistently indicate that curing with the zeolite/Fe_2_O_3_/Ca_3_(PO_4_)_2_ composite progressively redistributed PTEs from environmentally mobile fractions toward persistent geochemical pools.

### 3.5. Evidence-constrained interpretation of PTE stabilization mechanisms

The combined structural, surface-chemical, and fractionation results support a multicomponent stabilization framework rather than a single dominant mechanism. The progressive redistribution of potentially toxic elements (PTEs) from the more labile F1–F2 fractions towards less labile F3–F5 fractions provides the primary evidence of stabilization. The XPS comparison before and after curing (Fig. 19) further provides surface-sensitive evidence of changes in the near-surface chemical environment of the Fe_2_O_3_-modified zeolite. Together, these observations are consistent with contributions from ion exchange, interactions with Fe-containing surfaces, and phosphate-associated processes, with the observed fractionation response varying among PTEs and over the curing period.

**Fig. 19:**
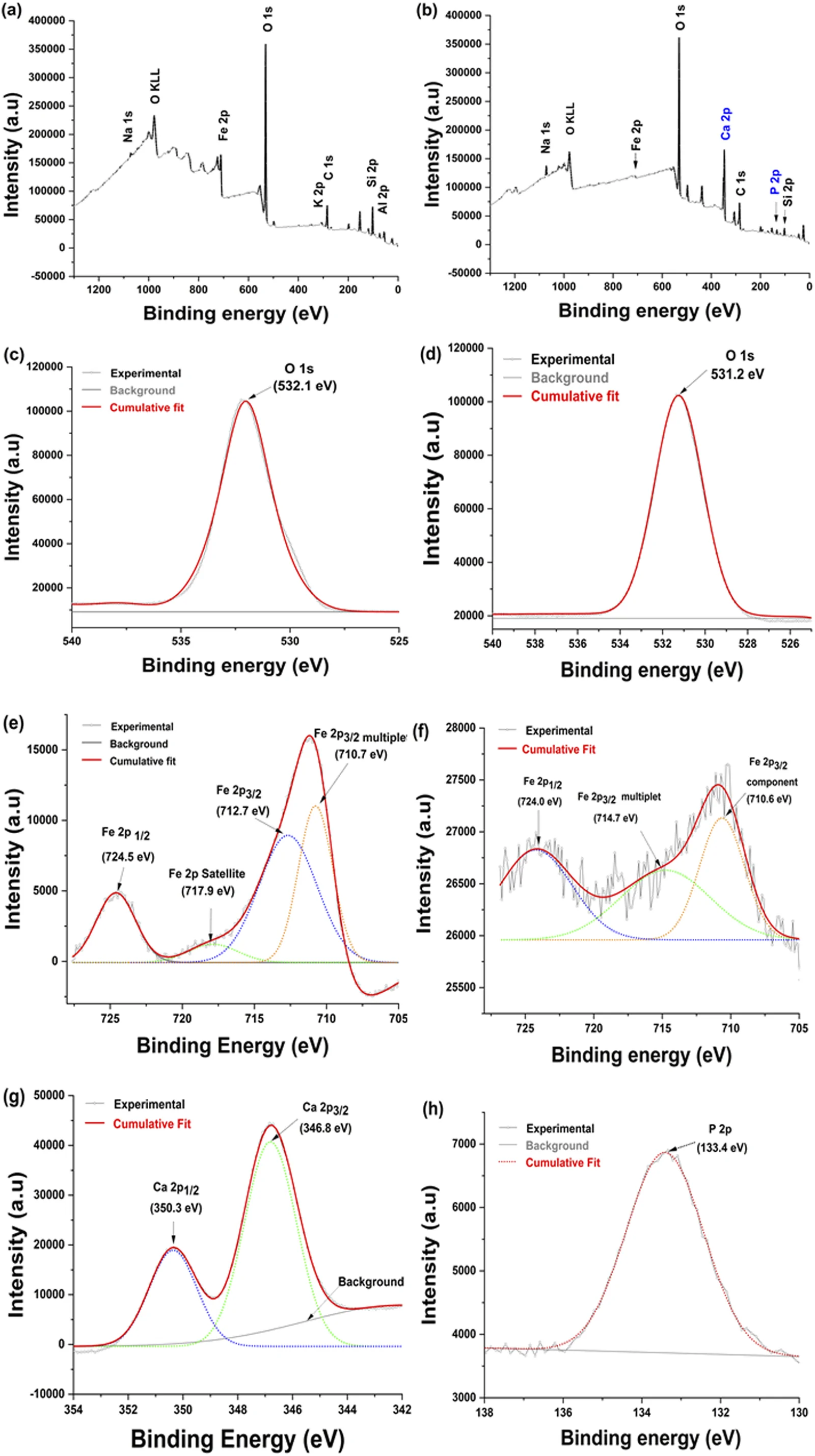
XPS characterization of the material before and after curing: (a and b) survey XPS spectra before and after curing, respectively; (c and d) high-resolution O 1s spectra before and after curing, respectively; (e and f) high-resolution Fe 2p spectra before and after curing, respectively; and (g and h) high-resolution Ca 2p and P 2p spectra, respectively, after curing.

The increase in pH from 5.6 to approximately 7.7 during curing likely altered the physicochemical environment governing PTE partitioning. Near-neutral conditions can favour the retention of several metal species by modifying aqueous speciation, mineral-surface charge, and the stability of metal-bearing phases.^47,48^ Similar pH-dependent effects have been reported for iron-oxide-containing stabilization systems.^39^ For hematite surfaces, changes in pH can alter the protonation state of surface hydroxyl groups and, consequently, the surface charge and affinity for dissolved species. The shift toward near-neutral conditions may therefore have modified the availability of Fe-associated surface sites during curing, although the present data do not permit the magnitude or direction of these surface-charge changes to be quantified directly. The concurrent changes in PTE fractionation, particularly for Pb and Cd, are consistent with progressive redistribution towards less labile forms. Nevertheless, the present results do not allow the contribution of pH to be separated from those of the amendment components and curing-induced changes in the solid matrix.

Natural zeolite provides an initial retention pathway through its negatively charged aluminosilicate framework, which can facilitate cation exchange with dissolved metal species.^10^ Surface oxygen-containing groups may provide additional sites for PTE association, although their specific contribution cannot be resolved from the present dataset.^11,12^ The comparatively limited redistribution observed in the natural-zeolite-amended system suggests that zeolite-mediated processes contribute primarily to the retention of more readily exchangeable PTE pools. However, because Ca_3_(PO_4_)_2_ was also present in this treatment, its contribution cannot be independently separated from that of the zeolite.

Fe_2_O_3_ modification introduces additional Fe-containing interfacial environments. The XRD, FTIR, BET, and SEM-EDS results establish the structural and textural modification of the zeolite, while the XPS spectra provide complementary surface-chemical evidence. Before curing, the Fe 2p spectrum exhibits the characteristic Fe 2p doublet and multiplet structure consistent with Fe^3+^-containing iron-oxide surface species (Fig. 19e). After curing, changes in the Fe 2p profile, including the fitted components at approximately 710.6, 714.7, and 724.0 eV (Fig. 19f), indicate an altered near-surface chemical environment following interaction with the bottom-ash matrix. Such changes are consistent with modification of Fe-containing interfacial environments during curing. Surface adsorption and ligand-exchange processes have been reported for Fe-containing mineral interfaces^33,39^ and may therefore represent plausible retention pathways in the present system. Similar enhanced retention has been reported for Fe-modified zeolitic materials, where Fe-rich interfaces may provide additional binding environments beyond those of the parent zeolite.^15^ However, the present XPS data do not establish a specific PTE–Fe coordination configuration.

The absence of further stabilization improvement at 12 wt% Fe_2_O_3_, together with the observed BET characteristics, indicates that increasing Fe_2_O_3_ loading did not necessarily translate into greater stabilization performance within the tested range. This behaviour can be interpreted within a loading-dependent structure–property framework, in which increasing the amount of an active component does not necessarily produce a proportional performance gain when surface accessibility and pore structure become limiting factors. A recent study of an Fe-containing multifunctional composite similarly reported that an intermediate composition provided a more favourable balance between reactive sites and accessible pore structure, whereas excessive incorporation of one component was associated with particle aggregation and reduced pore accessibility.^49^ Although the material system and target pollutants differed from those investigated here, this recent evidence provides useful broader context for interpreting the present non-monotonic response to Fe_2_O_3_ loading. The specific cause of the plateau at 12 wt% Fe_2_O_3_ remains unresolved, and the present results therefore support a loading-dependent relationship rather than a simple monotonic dependence on total Fe_2_O_3_ content.

The XPS comparison provides complementary evidence for the presence of Ca- and P-containing surface species after curing. Ca 2p and P 2p signals are evident after curing (Fig. 19g and h). Combined with the pronounced redistribution of Pb and Cd towards less labile fractions, these observations are consistent with a contribution from phosphate-associated retention and/or precipitation processes during curing. The greater redistribution of Pb and Cd than Zn and Cu may reflect differences in their interactions with phosphate-bearing phases, consistent with previous reports of sparingly soluble phosphate-bearing phases involving Pb and Cd under favourable conditions.^31,41^ Related apatite- and phosphate-assisted stabilization systems have likewise demonstrated the potential of phosphate-bearing amendments to promote the transfer of PTEs towards less labile solid-phase associations.^19,39,50^ Nevertheless, the present XPS and XRD data do not directly identify the structure, crystallinity, or specific composition of PTE-bearing phosphate phases. Phosphate-associated precipitation should therefore be regarded as a plausible contributing pathway rather than a directly demonstrated mechanism.

The Fe 2p profile after curing was fitted with the main Fe 2p_3/2_ and Fe 2p_1/2_ contributions together with a multiplet-related feature at approximately 714.7 eV (Fig. 19f). This feature was treated as a multiplet contribution within the Fe 2p_3/2_ envelope rather than as evidence of an independent Fe chemical state. The corresponding fitting parameters, including binding energy, FWHM, and relative area, are provided in Table S5 (SI).

The O 1s envelope also changes after curing, with its dominant position shifting from approximately 532.1 to 531.2 eV (Fig. 19c and d), indicating a change in the overall near-surface oxygen environment. Together with the evolution of the Fe 2p profile and the emergence of Ca 2p and P 2p signals, this shift is consistent with changes at the amendment–bottom-ash interface during curing. Because the O 1s contributions are strongly overlapping, the shift is not assigned to a specific oxygen species or used for quantitative component analysis. These surface-sensitive changes complement the bulk structural information obtained from XRD and FTIR and support the occurrence of chemical and structural evolution during curing.

PTE-specific behaviour further supports a chemically heterogeneous stabilization response. Pb and Cd underwent the most pronounced redistribution towards F5, indicating greater partitioning into the operationally defined less labile fractions. As and Cr exhibited fractionation responses distinct from those of the predominantly cationic PTEs. Their behaviour cannot be interpreted solely in terms of zeolitic cation exchange and may involve interactions with Fe-containing surfaces and other components of the bottom-ash–amendment matrix. Such interactions are consistent with the reported reactivity of Fe-rich mineral interfaces towards oxyanion species,^51,52^ although the present data do not resolve the oxidation states or specific surface associations of As and Cr. The observed redistribution of Cr is also broadly consistent with previous studies of Cr stabilization by Fe-bearing materials,^53,54^ although the present data do not establish the specific surface complexes or Cr-bearing phases involved. Zn showed a slower shift towards less labile fractions, whereas Cu exhibited limited redistribution because a substantial proportion was already associated with the residual fraction before treatment. The PCA results further captured these differences in fractionation patterns, with Pb and Cd showing greater displacement from the F1–F2-associated region, whereas Zn and Cu exhibited comparatively smaller positional shifts during curing.

Overall, the proposed framework (Fig. 20) describes stabilization as a coupled structure–surface–fractionation process.

**Fig. 20:**
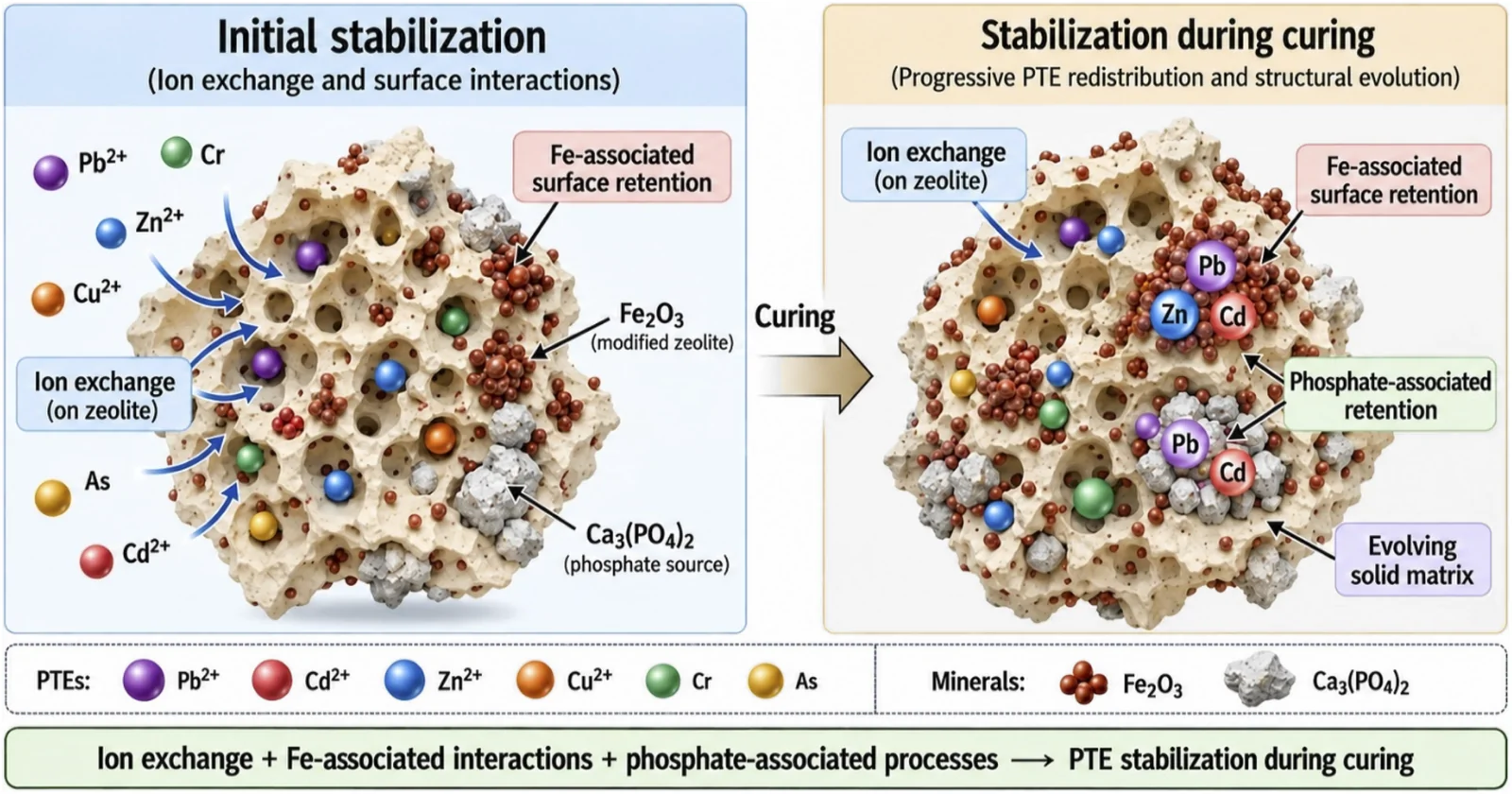
Evidence-constrained framework for PTE stabilization by the natural zeolite/Fe_2_O_3_/Ca_3_(PO_4_)_2_ composite during curing.

Natural zeolite provides a matrix for exchange and surface retention; Fe_2_O_3_ introduces additional Fe-containing interfacial environments; and Ca_3_(PO_4_)_2_ provides a phosphate source that may contribute to further PTE retention. The observed redistribution of PTEs towards less labile fractions, together with the structural, physicochemical, multivariate, and XPS evidence, supports this integrated interpretation. However, the present dataset does not permit adsorption, surface complexation, ion exchange, Fe-associated retention, and phosphate-associated precipitation to be individually resolved. The proposed framework should therefore be regarded as an evidence-constrained interpretation of PTE stabilization under the investigated four-week curing conditions, rather than a definitive assignment of molecular-scale mechanisms or long-term stability.

### 3.6. Integrated interpretation of PTE stabilization during curing

The integrated results address the three questions posed in the Introduction: how PTEs are redistributed among operationally defined fractions, how Fe_2_O_3_ loading relates to amendment structure and PTE partitioning, and how stabilization evolves during curing. Collectively, the structural, physicochemical, fractionation, and multivariate evidence supports a coupled structure–fractionation–time framework for the amended MSWI bottom ash system.

First, stabilization was characterized by progressive redistribution of PTEs from the more labile F1–F2 fractions towards less labile fractions, including F5, although the magnitude of redistribution was element dependent. Pb and Cd showed more pronounced shifts than Zn and Cu. These trends reflect changes in operationally defined PTE partitioning but do not resolve the molecular identity of the retained species or distinguish among sorption, complexation, ion exchange, and precipitation mechanisms.

Second, Fe_2_O_3_ loading was associated with both structural evolution of the modified zeolite and PTE fractionation. Within the investigated range, 8 wt% Fe_2_O_3_ provided the best stabilization performance, whereas increasing the loading to 12 wt% yielded no further improvement. The lack of further improvement at 12 wt% suggests that higher Fe_2_O_3_ loading did not enhance stabilization within the tested range, although the underlying cause remains unresolved. Thus, the results support an Fe_2_O_3_-loading-dependent relationship between amendment structure and PTE partitioning rather than a simple monotonic increase in stabilization with Fe_2_O_3_ content.

Third, stabilization evolved progressively during the four-week curing period. The concurrent changes in pH and EC, together with the evolving PTE fractionation patterns characterized by PCA and HCA, indicate continuing physicochemical and partitioning changes rather than a static amendment effect. Collectively, these findings link amendment structure and Fe_2_O_3_ loading with PTE partitioning, while the temporal analyses demonstrate its progressive evolution during curing.

Overall, the findings support a structure–fractionation–time framework for interpreting PTE stabilization under the investigated conditions. This framework is supported by convergent structural, physicochemical, fractionation, and multivariate evidence, while molecular-scale mechanisms and longer-term stability remain unresolved without direct spectroscopic, phase-specific, or aging evidence.

### 3.7. Leachability of PTEs under aqueous and simulated acid-rain conditions

The batch-leaching experiments directly quantified the release of PTEs from untreated and stabilized bottom ash under deionized-water (DI) and mildly acidic extraction conditions. Stabilization decreased the leachable concentrations of all six investigated PTEs, although the magnitude and statistical significance of the reductions varied among elements. Detailed leaching data and statistical analyses are provided in Tables S6 and S7 (SI). These results complement the Tessier sequential extraction by providing an independent measure of PTE release under defined extraction conditions.

Under DI-water extraction, leachable concentrations of As, Cr, Cd, Pb, Zn, and Cu decreased from 0.418, 1.56, 0.463, 2.53, 0.789, and 1.45 mg kg^−1^ in untreated bottom ash (T1) to 0.163, 0.816, 0.136, 0.651, 0.334, and 0.545 mg kg^−1^, respectively, after stabilization (T2) (Table S6 (SI)). The corresponding apparent immobilization efficiencies, calculated as described in Section 2.5, were 61.0%, 47.8%, 70.6%, 74.3%, 57.6%, and 62.5%, respectively.

The corresponding apparent immobilization efficiencies were 61.0%, 47.8%, 70.6%, 74.3%, 57.6%, and 62.5%, respectively. Pb and Cd exhibited the largest reductions, whereas Cr showed the smallest. The reductions in As, Cr, Cd, Pb, and Cu were statistically significant, whereas the decrease in Zn was not (*p* = 0.098).

Simulated acid-rain extraction resulted in higher leachable concentrations of all six PTEs than DI-water extraction, indicating enhanced PTE release under the mildly acidic conditions (Table S6 (SI) and Fig. 21).

**Fig. 21:**
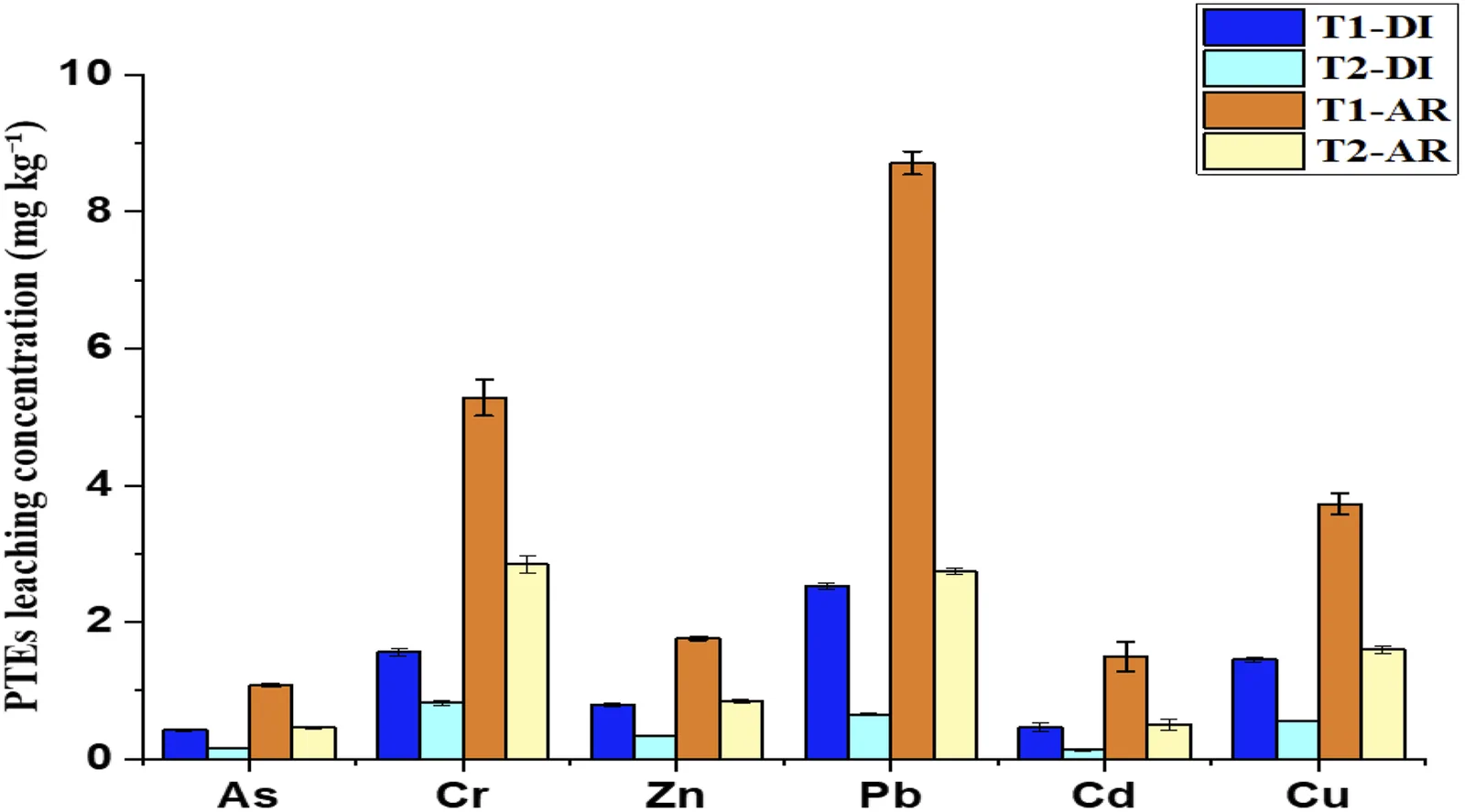
Leachable concentrations of As, Cr, Cd, Pb, Zn, and Cu in untreated (T1) and stabilized (T2) bottom ash under deionized-water (DI) and simulated acid-rain (AR) extraction conditions. T2 denotes bottom ash stabilized with zeolite/8 wt% α-Fe_2_O_3_/Ca_3_(PO_4_)_2_ for four weeks. Values are mean ± SD (*n* = 3).

Relative to T1-DI, concentrations in T1-AR increased by approximately 2.23–3.44-fold, while those in T2-AR increased by 2.55–4.20-fold relative to T2-DI. Despite the enhanced release, T2 consistently exhibited lower leachable concentrations than T1 for all six PTEs. The corresponding apparent immobilization efficiencies were 57.3% for As, 46.2% for Cr, 66.3% for Cd, 68.6% for Pb, 51.7% for Zn, and 57.1% for Cu. The lower efficiencies under simulated acid-rain conditions indicate that PTE retention was less effective under acidic extraction than under DI-water conditions, although substantial reductions in leachable PTE concentrations were maintained.

The leachate chemistry also differed between untreated and stabilized bottom ash. Under DI conditions, pH increased from 6.21 ± 0.024 in T1 to 7.93 ± 0.043 in T2, while EC increased from 2.83 ± 0.017 to 3.29 ± 0.036 mS cm^−1^. Under simulated acid-rain conditions, pH increased from 5.803 ± 0.025 to 7.65 ± 0.042, whereas EC increased from 2.42 ± 0.008 to 2.78 ± 0.016 mS cm^−1^. The increase in leachate pH following stabilization may have contributed to PTE retention by altering aqueous speciation, mineral-surface interactions, and the solubility of PTE-bearing phases. This effect may be particularly relevant to As and Cr, whose aqueous speciation and mineral-surface interactions can vary with pH. However, the present data do not permit the contribution of pH to be quantitatively separated from that of the stabilization treatment. Notably, the reductions in As and Cr leachability were maintained under both DI-water and simulated acid-rain extraction, although their apparent immobilization efficiencies decreased from 61.0% to 57.3% for As and from 47.8% to 46.2% for Cr under the more acidic extraction conditions. These observations, together with the progressive redistribution of As and Cr from the labile F1–F2 fractions toward less labile fractions during curing, are consistent with contributions from both the extraction environment and changes in PTE partitioning. Overall, the data support improved As and Cr retention following stabilization, while indicating that retention remained sensitive to the extraction conditions.

Statistical analysis further confirmed significant treatment-related reductions for five of the six PTEs under DI-water extraction and for all six PTEs under simulated acid-rain conditions (Table S7 (SI)). The stronger reductions observed for Pb and Cd are consistent with their pronounced redistribution towards less labile fractions in the sequential extraction results, whereas the comparatively smaller reduction for Cr is consistent with its more limited stabilization response. These findings demonstrate that the reduction in leachable PTE concentrations was element dependent and varied between extraction conditions.

Collectively, the batch-leaching results provide an independent line of evidence for reduced PTE release following stabilization, complementing the redistribution observed in the Tessier sequential extraction. Whereas sequential extraction characterizes operationally defined F1–F5 partitioning, batch leaching quantifies PTE release under specified aqueous and mildly acidic conditions. The concurrent decrease in leachable concentrations and shift towards less labile fractions therefore strengthens the evidence for reduced PTE mobility after stabilization. However, these results are restricted to the tested extraction conditions and do not establish long-term environmental stability. The 24 h extraction of four-week-cured material does not reproduce prolonged weathering, repeated wetting–drying, redox fluctuations, or field-scale leaching. Long-term PTE persistence and potential remobilization under environmental ageing conditions therefore remain to be established.

### 3.8. Study limitations and future perspectives

The present study evaluated PTE stabilization primarily through changes in their operationally defined partitioning among F1–F5 during four weeks of curing. The observed redistribution from the more labile F1–F2 fractions towards less labile F3–F5 fractions supports stabilization under the investigated conditions but does not establish long-term environmental durability or irreversible retention. The additional batch-leaching experiments provided evidence of reduced PTE release under deionized-water and simulated acid-rain conditions; however, these results remain specific to the tested extraction conditions and do not address long-term persistence or secondary remobilization under environmental ageing.

A further limitation concerns component-specific attribution of the stabilization response. Because Ca_3_(PO_4_)_2_ was maintained as a constant component of the amended systems, its individual contribution could not be independently quantified. The respective contributions and potential interactions of zeolite, Fe_2_O_3_, and Ca_3_(PO_4_)_2_ therefore cannot be quantitatively separated from the present experimental design. The results support a multicomponent stabilization framework rather than a quantitatively demonstrated synergistic effect. Component-resolved controls, preferably within a factorial design, would allow the main and interaction effects of individual amendment components to be distinguished. The limited number of Fe_2_O_3_ loading levels also precludes precise identification of the performance optimum and limits resolution of the loading–performance relationship. Future optimization could therefore begin with approximately 2 wt% increments across the 4–12 wt% range, followed by finer 1 wt% spacing around 8 wt%. This design would help determine whether the observed optimum is broad or local.

The molecular- and mineral-scale mechanisms also remain incompletely resolved. Although XPS, XRD, FTIR, SEM-EDS, and sequential extraction provide complementary evidence, they do not unambiguously resolve the specific processes or PTE-bearing phases responsible for retention. Desorption experiments, XAS, and HR-TEM could provide further evidence regarding retention reversibility, local coordination environments, and nanoscale phase formation.

The batch-leaching assessment demonstrates reduced PTE release under the investigated aqueous and mildly acidic conditions but does not represent a standardized regulatory leaching test or long-term environmental ageing. Future studies should therefore combine standardized leaching protocols with accelerated ageing involving variable pH and redox conditions, wetting–drying cycles, and prolonged weathering to evaluate PTE remobilization and stabilization persistence over extended periods.

Finally, the conclusions are specific to the investigated clinoptilolite-based Fe_2_O_3_/Ca_3_(PO_4_)_2_ system and MSWI bottom ash. Extension of the structure–fractionation–time framework to other zeolites should consider their framework mineralogy, cation-exchange capacity, surface area and pore structure, Fe speciation/loading, and chemical stability. For other waste matrices, key considerations include pH and buffering capacity, major-element and mineralogical composition, PTE concentrations and fractionation, and redox and curing conditions. These factors govern, or may modify, PTE partitioning, reactive-site availability, and the stability of PTE-bearing phases. The framework should therefore be viewed as a transferable conceptual workflow rather than a universal predictive model, with applicability to other zeolite-waste systems requiring comparative characterization and validation.

## 4. Conclusions

The stabilization of potentially toxic elements (PTEs) in MSWI bottom ash was associated with the coupled evolution of amendment structure and PTE fractionation during curing. The zeolite/α-Fe_2_O_3_/Ca_3_(PO_4_)_2_ system promoted redistribution of PTEs from the more labile F1–F2 fractions towards less labile F3–F5 fractions, although the extent of stabilization was element dependent.

Among the investigated Fe_2_O_3_ loadings, 8 wt% provided the best overall stabilization performance within the tested range, whereas increasing the loading to 12 wt% produced no further improvement. Overall stabilization followed the sequence Pb > Cd > As > Cr > Zn > Cu. The combined structural, surface-chemical, and fractionation evidence supports a multicomponent stabilization framework involving zeolite-mediated ion exchange, Fe-associated surface interactions, phosphate-associated processes, and progressive structural and textural evolution. However, the present experimental design does not permit their individual contributions or interaction effects to be quantitatively resolved.

Collectively, the results support a structure–fractionation–time framework for interpreting PTE stabilization under the investigated curing conditions. This framework links Fe_2_O_3_ loading and amendment evolution with time-dependent PTE redistribution while distinguishing experimentally supported fractionation changes from unresolved molecular-scale mechanisms. Further component-resolved experiments, standardized regulatory leaching tests, and long-term ageing studies are required to evaluate stabilization persistence, mechanistic contributions, and broader applicability.

## Author contributions

Thi Thu Thuy Nguyen engaged in the conceptualization, methodology, and manuscript preparation. Truong Xuan Vuong contributed significantly to data interpretation and writing and editing manuscript. Thi Thu Ha Pham, Minh Binh Tu, Dinh Vinh Nguyen, Thi Viet Pham, Thi Giang Ngo, Anh Quoc Hoang, Thi Hue Nguyen, Xuan Hoa Vu, Tran Thu Trang, Xuan Thang Dam contributed equally to data collection, analysis, and manuscript review. All authors approved the final version of the manuscript.

## Conflicts of interest

The authors declare no conflicts of interest.

## Supplementary Material

RA-OLF-D6RA06895B-s001

## Data Availability

All data supporting the findings of this study are available within the article and its supplementary information (SI). Supplementary information: experimental design and composition of the stabilization mixtures (Table S1), analytical quality assurance and quality control (QA/QC) data for the ICP-OES measurements, including accuracy, precision, method detection limits (MDLs), limits of quantification (LOQs), and recovery using certified reference materials (Tables S2 and S3), as well as the complete sequential extraction results for As, Cr, Zn, Pb, Cd, and Cu in untreated and treated municipal solid waste incineration bottom ash after curing (Table S4). These SI data support the experimental methodology, analytical validation, chemical-speciation analysis, and interpretation of the results presented in the main manuscript. See DOI: https://doi.org/10.1039/d6ra06895b. Additional experimental data and supporting information are available from the corresponding author upon reasonable request.
