# Supplementary material for: Linking α-Fe2O3-modified zeolite, amendment structure, and PTE fractionation during municipal solid waste incineration bottom ash stabilization
Source: RSC Adv. 2026 Oct 8;16(46):60931–59. doi: 10.1039/d6ra06895b (PMC13647311; doi:10.1039/d6ra06895b)
Supplement: Supplementary file 1 [file RA-016-D6RA06895B-s001.pdf]

Supplementary Information (SI) for RSC Advances.
This journal is © The Royal Society of Chemistry

Supplementary Information

Title: Linking α-Fe₂O₃-Modified Zeolite, Amendment Structure, and PTE Fractionation during Municipal Solid Waste Incineration Bottom Ash Stabilization

Manuscript ID: RA-ART-07-2026-006895. R1

Table S1. Experimental design and composition of stabilization mixtures used for heavy-metal immobilization of municipal solid waste incineration bottom ash.

Sample	Treatment	Natural zeolite (g)	Fe₂O₃ loading (wt%)	Ca₃(PO₄)₂ (g)	Bottom ash (g)
M0	Control	–	–	–	**2.0**
M1	Zeolite + Ca₃(PO₄)₂	0.5	0	0.2	2.0
M2	Zeolite/Fe₂O₃ (4 wt%) + Ca₃(PO₄)₂	0.5	4	0.2	2.0
M3	Zeolite/Fe₂O₃ (8 wt%) + Ca₃(PO₄)₂	0.5	8	0.2	2.0
M4	Zeolite/Fe₂O₃ (12 wt%) + Ca₃(PO₄)₂	0.5	12	0.2	2.0

Table S2. Accuracy and precision of the ICP–OES method evaluated using the certified reference material MESS-4 (n = 3).

Element	Replicate 1	Replicate 2	Replicate 3	Measured value (Mean ± SD, mg kg^{-1})	RSD (%)	Certified value (mg kg^{-1})	Recovery (%)
As	32.3	35.4	33.7	33.8 ± 2.1	4.59	32.9 ± 1.8	102.7
Cd	21.9	23.7	23.1	22.9 ± 1.1	3.64	21.5 ± 1.2	106.2
Cu	23.1	21.2	22.7	22.3 ± 1.4	4.49	21.7 ± 2.8	102.9
Cr	0.263	0.253	0.306	0.274 ± 0.038	9.69	0.280 ± 0.040	97.5
Pb	156	142	162	153 ± 13.9	6.69	147 ± 6.0	104.3
Zn	84.9	86.1	90.2	87.1 ± 3.8	3.21	94.3 ± 1.8	92.3

Table S3. Repeatability of ICP–OES measurements for a multi-element standard solution (n = 10).

Replicate	As	Cd	Cu	Cr	Pb	Zn
1	0.249	0.254	0.249	0.243	0.235	0.262
2	0.261	0.246	0.270	0.261	0.260	0.244
3	0.261	0.241	0.253	0.283	0.240	0.267
4	0.247	0.244	0.275	0.257	0.234	0.259
5	0.258	0.274	0.267	0.271	0.243	0.269
6	0.263	0.257	0.246	0.276	0.233	0.258
7	0.254	0.242	0.256	0.266	0.250	0.244
8	0.257	0.258	0.242	0.254	0.228	0.253
9	0.246	0.261	0.253	0.258	0.244	0.271
10	0.276	0.246	0.249	0.256	0.235	0.259

Replicate	As	Cd	Cu	Cr	Pb	Zn
Mean ± SD (mg L^{-1})	0.257 ± 0.009	0.252 ± 0.010	0.256 ± 0.011	0.262 ± 0.012	0.240 ± 0.010	0.259 ± 0.010
RSD (%)	3.46	4.13	4.30	4.47	4.00	3.67
MDL (mg L^{-1})	0.0268	0.0313	0.0329	0.0350	0.0288	0.0285
LOQ (mg L^{-1})	0.0894	0.1042	0.1098	0.1165	0.0960	0.0949
Mean/MDL	9.60	8.07	7.77	7.51	8.33	9.08

Table S4. Distribution of metal fractions in ash samples (M0–M4) after incubation.

Sample	Chemical fraction(s)	Concentration (mg/kg)					
		As	Cr	Zn	Pb	Cd	Cu
M0	F1	18.58	21.68	480.4	2.469	8.900	490.0
	F2	15.65	16.35	1325	43.59	23.41	882.6
	F3	25.34	30.09	737.5	35.09	22.56	2091
	F4	30.98	28.35	1338	21.60	24.15	1210
	F5	36.90	452.9	1335	61.41	40.48	855.4
	SUM	127.4	549.4	5215	164.2	119.5	5529
M1	F1	18.24	18.63	453.1	2.11	7.684	444.3
	F2	15.37	15.28	1345	44.76	24.02	846.5
	F3	22.53	31.24	759.6	35.55	22.87	2090
	F4	33.26	26.55	1327	24.6	23.59	1191
	F5	42.88	476.5	1497	66.36	49.05	1100
	SUM	132.3	568.2	5382	173.4	127.2	5671
	H%	103.8	103.42	103.2	105.6	106.5	102.6
M2	F1	11.56	14.04	311.3	1.86	4.676	398.9
	F2	15.01	11.43	1302	36.50	18.29	787.2
	F3	25.08	33.72	792.2	37.30	23.95	2154
	F4	31.80	24.13	1274	21.46	23.47	1211
	F5	47.16	432.7	1841	75.9	51.56	1290
	SUM	130.6	516.0	5520	173.0	121.9	5841
	H%	102.5	93.92	105.8	105.4	102.0	105.6
M3	F1	4.945	11.772	191.9	0.8522	1.928	273.4
	F2	9.380	9.050	1185	24.48	13.43	679.0
	F3	35.28	36.47	800.1	38.67	25.42	2276
	F4	24.79	23.69	1298	20.26	23.10	1178

	F5	63.64	462.7	1954	89.74	59.74	1410
	SUM	138.0	543.7	5429	174.0	124.1	5817
	H%	108.3	99.0	104.1	106.0	103.5	105.2
M4	F1	5.432	12.28	264.8	1.460	2.534	290.0
	F2	11.97	9.438	1127	25.31	14.94	696.5
	F3	27.98	32.08	690.5	35.44	23.27	2181
	F4	26.57	29.11	1312	21.8	26.75	1184
	F5	58.14	479.6	1901	86.26	54.25	1344
	SUM	130.1	562.5	5295	170.3	122.3	5696
	H%	102.1	102.4	101.5	103.7	101.9	103.0

M0: untreated ash; M1: ash + natural zeolite; M2: ash + zeolite/Fe₂O₃ (4%); M3: ash + zeolite/Fe₂O₃ (8%); M4: ash + zeolite/Fe₂O₃ (12%).

Table S5. XPS Fe 2p fitting parameters for the α-Fe₂O₃-modified zeolite composite after curing.

**Contribution**	**Binding energy (eV)**	**FWHM, w (eV)**	**Gaussian area A (a.u.·eV)**	**Relative area**
Fe 2p₃/₂ main component	**710.64**	**3.86**	**4819.7**	**30.03%**
Fe 2p₃/₂ multiplet-related feature	**714.77**	**7.73**	**5514.9**	**34.36%**
Fe 2p₁/₂	**724.22**	**6.21**	**5715.2**	**35.61%**
**Total**	—	—	**16049.8**	**100%**

Table S6. Leaching of PTEs from T1 and T2 under DI and simulated acid rain conditions

Parameter	T1-DI	T2-DI	Immobilization (%) -DI	T1-AR	T2-AR	Immobilization (%) - AR
pH	6.21 ± 0.024	7.93 ± 0.043	-	5.803 ± 0.025	7.65 ± 0.042	-
EC (mS cm^{-1})	2.83 ± 0.017	3.29 ± 0.036	-	2.42± 0.008	2.78 ± 0.016	-
As (mg kg^{-1})	0.418 ± 0.017	0.163 ± 0.008	61.0 ± 2.08	1.08 ± 0.023	0.461 ± 0.015	57.3 ± 0.671
Cr (mg kg^{-1})	1.56 ± 0.057	0.816 ± 0.037	47.8 ± 0.535	5.28 ± 0.267	2.84 ± 0.134	46.2 ± 0.262
Cd (mg kg^{-1})	0.463 ± 0.057	0.136 ± 0.019	70.6 ± 0.655	1.49 ± 0.216	0.506 ± 0.083	66.3 ± 0.658
Pb (mg kg^{-1})	2.53 ± 0.042	0.651± 0.023	74.3± 0.561	8.71 ± 0.168	2.74 ± 0.047	68.6 ± 0.978
Zn (mg kg^{-1})	0.789 ± 0.030	0.334 ± 0.008	57.6 ± 1.02	1.76 ± 0.021	0.851 ± 0.025	51.7 ± 1.45
Cu (mg kg^{-1})	1.45 ± 0.037	0.545 ± 0.006	62.5 ± 0.573	3.72± 0.150	1.59 ± 0.061	57.1 ± 0.202

Table S7. Two-way ANOVA and Tukey's pairwise comparisons of PTE leaching between T1 and T2 under DI and simulated acid rain (AR) conditions.

PTEs	p-value	Significance	p-value	Significance
	T1-DI/ T2-DI,		T1-AR/T2-AR,	
As	1.28 × 10⁻⁶	***	<0.001	***
Cr	0.00538	**	9.97 × 10⁻⁷	***
Cd	6.37 × 10⁻⁸	***	<0.001	***
Pb	2.33 × 10⁻⁸	***	<0.001	***
Zn	0.0980	a	1.57 × 10⁻⁴	***
Cu	2.03 × 10⁻⁵	***	<0.001	***

*a: p ≥ 0.05; *: p < 0.05; **: p < 0.01; ***: p < 0.001.*
